# An Automated Organotypic SCN Culture System Revealing Novel Insights into VIP Regulation of Circadian Rhythm

**DOI:** 10.1002/advs.202511069

**Published:** 2026-01-15

**Authors:** Kui Han, Meimei Liao, Jingpeng Zhang, Ruoyu Zhong, Long Mei, Dapeng Ju, Eric Erquan Zhang, Yanyi Huang

**Affiliations:** ^1^ Biomedical Pioneering Innovation Center (BIOPIC) Peking‐Tsinghua Center for Life Sciences College of Chemistry Peking University Beijing China; ^2^ Department of Anesthesiology The Second Affiliated Hospital Chongqing Medical University Chongqing China; ^3^ School of Life Sciences Peking University Beijing China; ^4^ Integrated Science Program, Yuanpei College Peking University Beijing China; ^5^ National Institute of Biological Sciences Beijing China; ^6^ Tsinghua Institute of Multidisciplinary Biomedical Research Tsinghua University Beijing China; ^7^ College of Chemistry and Molecular Engineering Beijing National Laboratory of Molecular Sciences Peking University Beijing China; ^8^ Shenzhen Bay Laboratory Institute for Cell Analysis Guangdong China

**Keywords:** circadian rhythm, ex vivo culture, PER2 reduction, suprachiasmatic nucleus

## Abstract

In mammals, the suprachiasmatic nucleus (SCN) functions as the primary driver of circadian rhythm. Although individual SCN neurons exhibit cell‐autonomous oscillations, intercellular communication—mediated by neural peptides such as vasoactive intestinal polypeptide (VIP)—synchronizes their rhythms. However, the precise molecular mechanism by which VIP facilitates SCN synchronization remains elusive. Here, we introduce an automated *ex vivo* culture system, termed ‘brain‐slice‐in‐a‐chamber’ (BaSIC), tailored for SCN slices, as well as dissociated cells culture. This apparatus automates medium exchange and ensures optimal control over temperature and humidity, ensuring a stable internal environment conducive to tissue culture. Furthermore, BaSIC enables real‐time observation of tissue responses to diverse but programmed stimuli. Using BaSIC, we demonstrate that VIP pulsing rapidly resets the circadian rhythm by synchronizing both phase and amplitude through a swift reduction of Period 2 (PER2) protein. Mathematical modeling, coupled with experimental validation, further suggests that VIP promotes the rapid reduction of PER2. Our findings, facilitated by BaSIC, provide new insights into SCN neuron synchronization, paving the way for advanced studies in chronobiology, with potential therapeutic applications for circadian disorders.

## Graphical abstract

An Automated ex vivo culture system BaSIC, tailored for SCN slices and cell culture, which automates medium exchange and ensures a stable internal environment is developed. BaSIC enables real‐time observation of tissue/cell responses to diverse but programmed stimuli. Using BaSIC, we demonstrate that VIP pulsing rapidly resets the circadian rhythm through a swift reduction of Period 2 (PER2) protein.

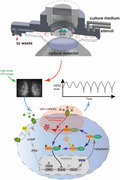

## 1 Introduction

Circadian cycles regulate a wide range of physiological processes, among which timing coordination is essential [1]. In mammals, among all internal clock systems, the suprachiasmatic nucleus (SCN), located in the hypothalamus, serves as the master clock, standing out due to its complex intercellular coupling mechanism, thus synchronizing the timing of peripheral clocks throughout the body [2, 3, 4, 5]. The SCN's internal functioning is governed by the transcription‐translation feedback loop (TTFL), where CLOCK/BMAL1 form the positive arm, while Period (PER1, PER2, PER3) and Cryptochrome (CRY1, CRY2) proteins constitute the negative arm. However, it is not merely the TTFL within single cells that sustains individual circadian function, but also intercellular coupling [6], which sustains robust synchronization among cells and is mainly regulated by neurotransmitters, such as vasoactive intestinal polypeptide (VIP) [7, 8], gamma‐aminobutyric acid (GABA) [9, 10, 11], and arginine vasopressin (AVP) [12, 13]. Recently, experimental and theoretical evidence [14, 15] suggests that the timing of VIP signaling plays a crucial role in determining the synchronization of SCN circadian rhythm. However, direct experimental evidence showing how the synchronization changes under the influence of timed VIP stimulations, even periodic VIP stimulations in continuous measurements, is still limited. Precisely controlling the timing, quantity, and frequency of neurotransmitter release to mimic physiological conditions by monitoring the circadian rhythms of the SCN network over extended periods is highly needed.

We have employed a microfluidic approach to examine the synthetic coupling dynamics of circadian rhythms, but that in vitro culture model was done at the cellular level [16]. For investigating the tissue‐level function of neurotransmitters, the ex vivo organotypic SCN culture (OSC) presents an ideal and highly informative model for elucidating the intricate molecular mechanisms driving circadian rhythms. Such a system provides a more physiological context than many liquid‐based cell cultures.

In this work, we hereby developed an automated *ex vivo* OSC culture apparatus, christened the ‘brain‐slice‐in‐a‐chamber’ (BaSIC), to offer high accuracy in controlling and assessing culture conditions throughout the entire culturing process over extended periods. BaSIC ensures a specific culturing condition where brain slices are maintained at the liquid‐gas interface to remain hydrated without being submerged in the medium, thereby offering a more realistic environment for studying its inherent rhythmicity [17] in the long‐term culture. BaSIC's modular design enables highly accurate and dynamic manipulation of culture environments, such as the application of periodic stimuli, through its programmable fluidic control system. It also facilitates continuous nutrient supply in a perfusion mode and precise temperature regulation, ensuring that SCN or cells remain in optimal state and are amenable to prolonged observation using optical measurement techniques for real‐time signal acquisition.

We employed BaSIC to optically monitor coupling responses by periodic and programmed VIP stimulations among neurons within SCN slices during *ex vivo* OSC. We found that high‐concentration VIP treatments led to a rapid decrease in bioluminescent signals of Period 2 luciferase fusion protein (PER2::LUC). Biochemical and cellular assays confirmed that the observed signal decrease was attributed to PER2 reduction, which caused circadian phase shifts and reset the clock mechanism. We also constructed a model that included our discovery to simulate experimental observations of neuronal circadian rhythms of SCN with VIP stimulation. Our result reveals that VIP not only enhances PER2 expression but also causes an immediate reduction of PER2. This paradoxical function of VIP effectively resets the circadian rhythm in SCN neurons to a synchronized baseline state, implying a regulatory pathway beyond the conventional TTFL gene network, and provides a more comprehensive understanding of the role of periodic neurotransmitter signaling in the synchronization and regulation of circadian rhythms.

## 2 Results

### 2.1 BaSIC, A Modular Device for Organotypic SCN Culture

**FIGURE 1:**
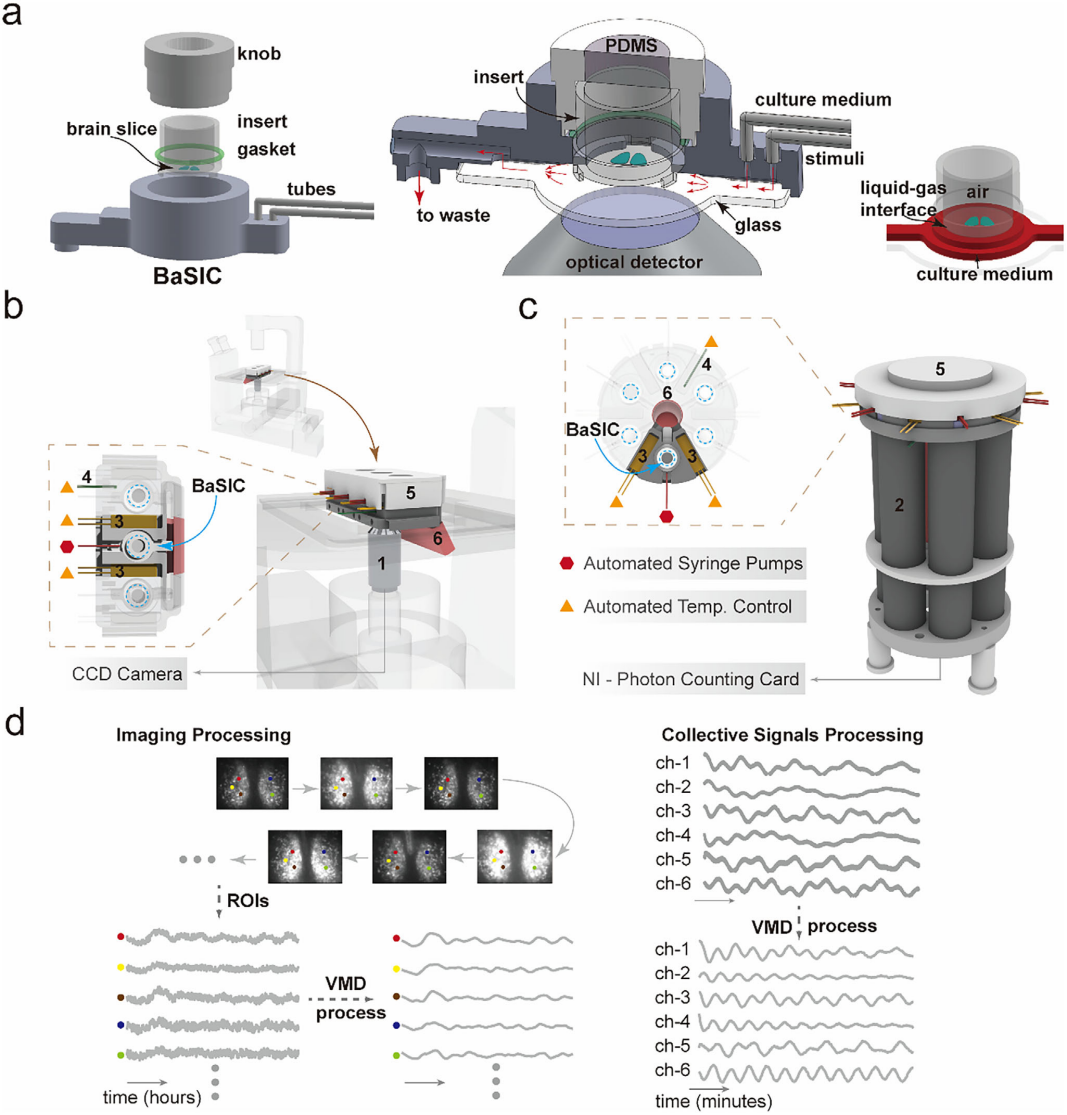
Schematic of brain‐slice‐in‐a‐chamber (BaSIC). a) BaSIC is a universal‐purpose Brain slice culture setup. The mouse brain slices can be placed on the semipermeable membrane of the insert and sealed in the chamber for long‐term culture. Two independent tubes are connected to BaSIC for material exchange, such as medium perfusion and stimuli. The bottom of BaSIC chamber is a glass window for imaging or acquiring other optical signals. A PDMS spacer is used to enable air exchange and keep the inner chamber sterilized. A semi‐open outlet ensures the minimum fluidic pressure in the flowing path, maintaining the constant level of the air‐liquid interface to facilitate a long‐term culture of brain slices. In b) and c) The BaSIC modules can be placed into different holders, and be integrated into a compact format for microscopic imaging, or be attached to the photo‐multiplier tubes for highly sensitive optical monitoring. Spatially resolved bioluminescent signals are captured using a CCD and an objective lens (1), and the collective bioluminescent signals are collected through a miniature PMT (2) and recorded through a photon counting card, respectively. Heating elements (3) and thermistors (4) are embedded in the holders to achieve a constant culture temperature at 37°C with automatic PID control. Water reservoirs (5) are placed on top of BaSIC units to provide constant humidity. The tanks (6) are used to collect waste liquids. d) Regions of interest in the image sequences are picked up, and the intensities are denoised by variational mode decomposition (VMD) and further analyzed to extract the circadian phases. The whole brain slice collective data are processed similarly.

We developed a modular BaSIC system (Figure 1a) to overcome the constraints posed by conventional static culture methods. The most critical advantage of BaSIC is avoiding recurrent manipulations of the Petri‐dish‐based experimental protocols during extensive culturing phases or drug screening tests, which inadvertently disrupts the rhythmic expression of circadian genes and thereby affects the integrity of data acquisition. A mouse SCN slice carrying circadian‐regulated reporters is delicately placed within a compact insert. The insert is then placed into the culture module, which is tightly sealed to eliminate possible environmental contaminations. Two independent tubes are used to separate the infusion of culture medium and the specific stimuli. Pressure‐regulated perfusion system is programmed to ensure accurate automation and coordination of flow, thereby preserving a stable microenvironment for slices.

In each BaSIC module, the SCN slice rests atop a membrane above a glass window to support high‐resolution optical imaging for intricate temporal and spatial studies, thereby enhancing the system's versatility in investigating complex brain slice cultures. Gas permeability was allowed by using a polydimethylsiloxane (PDMS) spacer while maintaining sterility (Figure 1a). The culture medium is meticulously controlled via syringe pump and dispensed into the chamber beneath the membrane. Meanwhile, the upper compartment retains an air‐filled environment, where the SCN slice is situated at an optimally maintained liquid‐gas interface, enhancing the survival rate of the long‐term culture of brain slices (Figure 1a). The system incorporates a semi‐open outlet for the medium flow, which reduces fluidic resistance and ensures unimpeded efflux.

The modular design of BaSIC guarantees accurate and stable long‐term control of temperature and humidity during tissue culture. These modules can be placed on the microscope stages for continuous imaging to capture high‐resolution spatiotemporal complexities of circadian rhythms or attached to photomultipliers (PMTs) to capture collective optical signals in parallel (Figure 1b,c; Figure S1). The signals are preprocessed with the variational mode decomposition (VMD) algorithm. Using the VMD algorithm, we decomposed the PER2 rhythm into components of different frequencies. We identified the lowest‐frequency component as the baseline, which reflects the average level of the PER2::LUC signal. By subtracting this baseline, we were able to normalize and compare PER2 rhythms across parallel experiments. (Figure 1d; Figure S2).

### 2.2 Spatial Correlation of Circadian Rhythms in SCN Explants with Timed VIP Pulses

We employed the BaSIC system to systematically study how VIP regulated the circadian phase in a periodical manner. To eliminate the impact of endogenous VIP secretion of SCN itself, *Vip* knockout SCN (*Vip‐/‐* SCN) carrying PER2::LUC [18] was employed in this study. Bioluminescent signals from PER2::LUC were recorded to reveal the expression pattern of PER2, thereby reflecting the circadian rhythm of SCN. The automated system allowed for the precise introduction of exogenous VIP pulses, periodically perfused once every 24 h. Between VIP pulses, a low‐flowing rate of culture medium was perfused for the remaining time, ensuring that the internal temperature remained unchanged and provided a constant supply of nutrients while minimally disturbing the culture environment within the BaSIC chambers.

**FIGURE 2:**
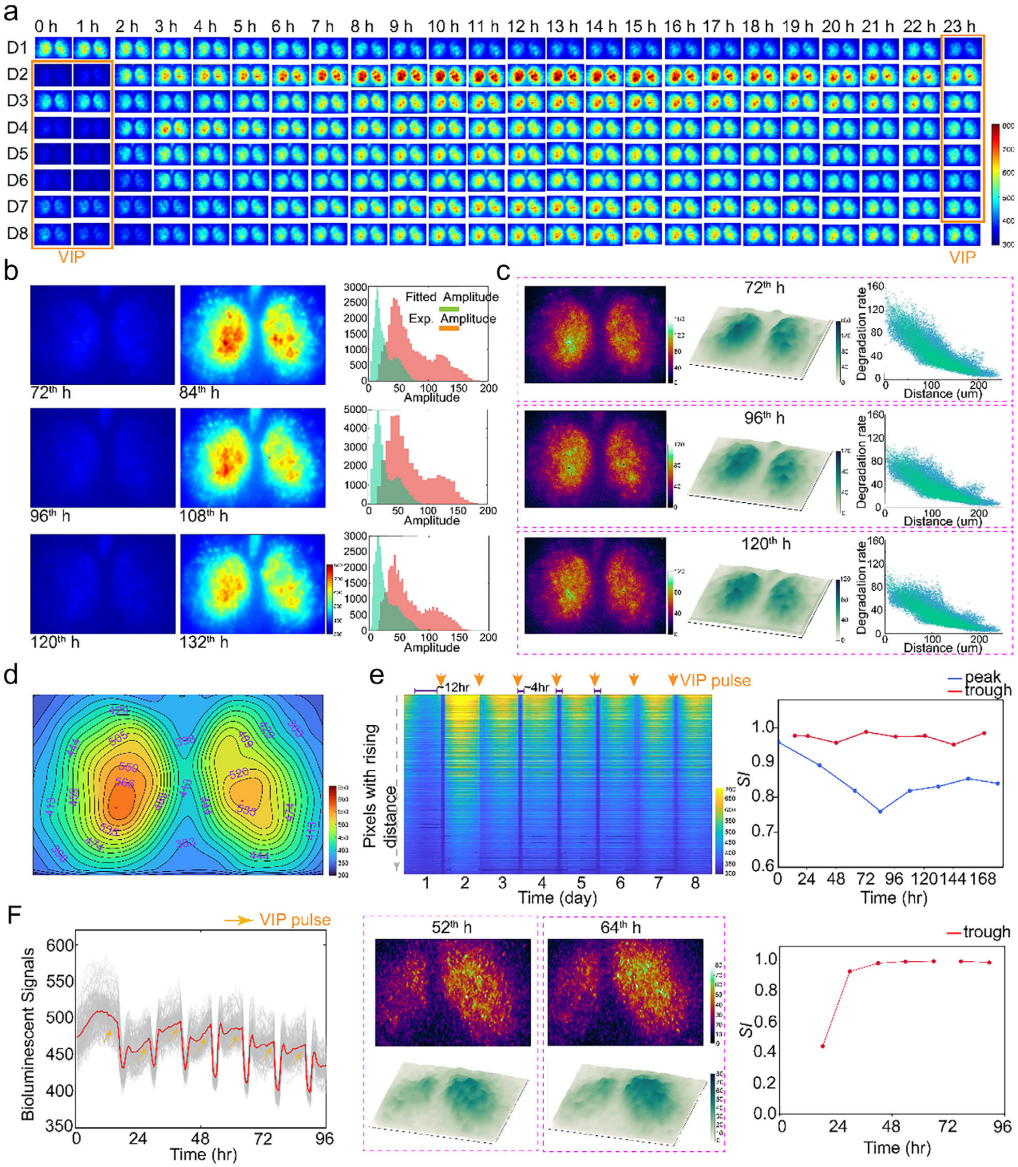
Spatial synchronization of amplitude and phase among SCN neurons. (a) 8‐circadian‐day time‐lapse bioluminescent images of an *Vip‐/‐* SCN slice. A regular 60 µM VIP introduction, with 24 h period and 2 h duration, was applied to the BaSIC culture chamber. Each VIP introduction was associated with a clear intensity drop (the trough). (b) Spatial distribution of PER2 during the phases of the troughs (left column) and the peaks (middle column) induced by VIP stimulations at the 72nd, 96th, and 120th hour. The histograms of amplitudes of VIP stimulated oscillations (experimental data in red, and sine‐function‐fitted data in green) are also presented. (c) Spatial distribution (left: 2D, middle: 3D) of the rate of PER2 reduction induced by VIP dosing at the 72th, 96th, and 120th hour. The scatter plots (right column) show the negative correlation between PER2 decrease rate and the distance from the centroids (black dots) of SCN slices (Pixels of the left area in blue, and pixels of the right area in green). (d) Spatial distribution of the average values of rhythm baselines that present basal expression levels of PER2 protein. The baselines are estimated from the lowest frequency of the VMD model. (e) Heatmap of PER2 rhythm of each pixel of SCN slice time‐lapse images. The data are sorted by distance from centroids, and VIP pulses are marked. Synchrony index (*SI*) was calculated for peak and trough, separately. (f) 60 µM VIP pulses stimulated an *Vip‐/‐* SCN slice with a 12‐h period. The grey curves depict the PER2::LUC oscillations from individual pixels of the *Vip‐/‐* SCN, while the red curve represents the averaged oscillation (left column). Orange arrows indicate the times at which VIP was added. Spatial distribution (upper: 2D, lower: 3D) of the rate of PER2 reduction induced by VIP dosing at the 52th, and 64th hour (middle column). Synchrony index was calculated for troughs (right column).

We utilized the BaSIC with a microscope (Figure 1b) to document the temporal progression of PER2::LUC luminescence through time‐lapse imaging and to reveal the neuronal coupling dynamics within the SCN. Unexpectedly, our results show that 60 µM VIP pulses robustly induce a synchronous response across all neurons, transiting momentarily to a dark state before quickly reverting to a bright state (Figure 2a). The collective change in luminescence corresponds directly to the rapid decrease in the overall PER2::LUC signals, suggesting that VIP might be orchestrating a form of transient resetting among SCN neurons. Further analysis of the temporal profiles of bioluminescence of individual pixels in the SCN region shows highly synchronized behavior (Figure S3). Signals are background‐subtracted through VMD algorithm.

The spatial distributions of PER2 after VIP induction (Figure 2b) exhibit a uniformly low level of PER2 protein with a nearly consistent trough value with insignificant peak alteration. We calculated the experimental amplitude of the PER2::LUC rhythm as the average of the absolute peak and trough values. Additionally, we fitted the rhythm data obtained under control conditions (without VIP stimulation) with a sine function, and defined the amplitude of the fitted sine wave as the fitted amplitude. The equation of the fitted sine function is provided below:

(1) $$\left[PER2\right]=\;a*\sin\left(\frac{2\pi }{24}t+b\right)+c.$$

The fitted amplitude was calculated using the same method for VIP stimuli administered at 72, 96, and 120 h. The histogram of amplitudes of oscillations (Figure 2b) shows that amplitudes are enhanced significantly by VIP stimulations. Subsequently, we divided the difference of bioluminescent signals between the initiation of the VIP pulse and the VIP pulse‐induced trough by the time interval between these two points to calculate the PER2 reduction rate, represented as V_PER2_. By V_PER2_, we quantitatively gauged the spatial heterogeneity in the rapid decrease of PER2 expression across the SCN. The spatial distribution of V_PER2_ illustrates a negative correlation between V_PER2_ and distance from the SCN's centroid (Figure 2c), which is consistent with previously reported findings [19] regarding the heterogeneous distribution of VIP receptor 2 (VIPR2) expression levels in SCN. These results indicate that cells in SCN with higher expression of VIPR2 and PER2 exhibit a greater PER2 reduction rate. The temporal average of PER2 signal also shows a similar pattern, with the baseline level of expression being higher around the SCN centroid (Figure 2d). These results indicate that neurons with initially elevated PER2 levels may experience more substantial reductions, playing a pivotal role in maintaining a highly precise synchronization across the entire neural network. The phase alignment (Figure 2e) demonstrates the temporal patterns of each pixel and shows the high degree of intraneuronal synchronization, especially with synchrony index [20, 21] (*SI*) approaching 1 at troughs, indicating that the rapid reduction of PER2 is not just an isolated phenomenon but rather part of a collective neuronal response. More interestingly, when the frequency of 60 µM VIP treatment is shortened by half, from the natural 24‐h circadian cycle to 12 h, it still induces a rapid decrease of PER2 signal (Figure 2f) with a similar spatial pattern. Further analysis of *SI* reveals a high degree of synchronization among cells during the troughs induced by VIP, demonstrating coherent oscillatory patterns at these nadirs. Conversely, PER2 signals at peaks exhibit a weaker synchronization, suggesting differential responsiveness or recovery phases among the cells. Our results indicate that SCN rhythmicity can be modulated by the frequency of VIP stimulation.

However, conventional Petri dish‐based SCN slices culturing and monitoring did not demonstrate rapid VIP‐induced PER2::LUC signal reduction under 60 µM VIP treatment (Figure S4), implying that extraneous influences in a conventional culture system may obscure the rapid decrease of PER2 signals.

### 2.3 Measurements of Circadian Rhythms With VIP Stimulations in BaSIC

**FIGURE 3:**
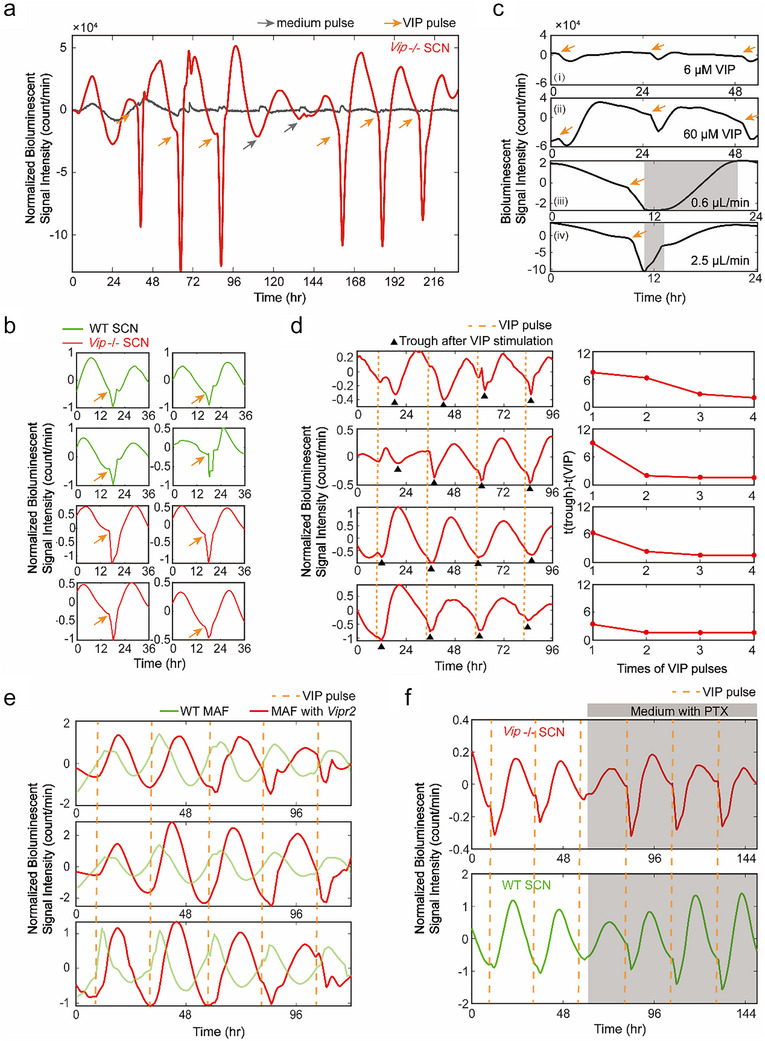
Rapid decrease of PER2::LUC signal and circadian resetting induced by periodic VIP pulses. (a) Circadian rhythm of *Vip‐/‐* SCN slices stimulated with 60 µM VIP. VIP was applied every 24 h period with 2 h duration. Between 96 and144 h, medium pulses replaced VIP pulses to simulate the condition with only culture medium (gray arrows). The gray curve represents the PER2‐LUC rhythmic curve of *Vip‐/‐* SCN with medium perfusion only. (b) Fast decrease of PER2 stimulated by 60 µM VIP dosing in multiple experiments using 4 wild‐type and 4 *Vip‐/‐* SCN slices. (c) PER2 rhythm of *Vip‐/‐* SCN slices stimulated by different concentrations of VIP and different elution rates. The shaded areas represent the duration of elution. VIP pulses are indicated by orange arrows. (d) PER2 rhythm of 4 *Vip‐/‐* SCN slices with 24‐h‐period VIP dosing at different phases of PER2 rhythm (left panel). Time intervals between the initial troughs and VIP pulses (right panel) become small and stable after several VIP pulses, indicating that the troughs of PER2 rhythm are reset to the time of VIP pulsing. (e) Circadian rhythm of wild‐type and VIP receptor over expression Mouse Adult Fibroblasts (MAF) cells stimulated with VIP (*n* = 3). 60 µM VIP was applied every 24 h. (f) PER2 rhythm of 1 wild‐type and 1 *Vip‐/‐* SCN slices with 1 µg/ml PTX introduction (shaded). VIP was perfused every 24 h with 2 h duration. VIP pulses are indicated by orange dashed lines.

We applied the collective signal measurement with multiple BaSICs (Figure 1b) to explore the possible mechanism of observed PER2 signal reduction by 60 µM VIP stimulations. The PER2::LUC bioluminescent signals were continuously recorded by PMTs for further processing. Consistently, we observed the rapid decrease in PER2::LUC bioluminescent signals immediately following pulses of exogenous VIP (Figure 3a), no matter when VIP was dosed at peak or trough phases of the circadian periods. Crucially, this VIP‐dependent PER2::LUC signal decline has no memory, in that the phenomenon occurs only while VIP is present and disappears once VIP is washed out. Once the VIP pulses are eliminated, the signal returns to its regular cycles. As a control, the result of *Vip‐/‐* SCN slices subjected only to medium pulse stimulations rules out potential confounding effects from temperature shock or serum shock [22]. Control experiments using Mouse Adult Fibroblasts (MAF) cell line expressing CMV‐driven luciferase (CMV‐LUC) were performed, and the results ruled out the possibility that the observed signal decrease was due to a direct interaction between the luciferase reporter and VIP (Figure S5). Both wild‐type (wt) SCN and *Vip‐/‐* SCN samples display a rapid decrease in PER2::LUC signals from multiple experiments (Figure 3b), suggesting that this unconventional response is not genotype‐specific. Additionally, we calculated the half‐life of PER2 during VIP application. By fitting the PER2 decay curve with an exponential function (Equation 2), we determined the half‐life (*t*_1/2_) of PER2 (Equation 3), which averaged 0.9 h across multiple SCN slices.

(2) $$\left[PER2\right]=\;a*e^{b*t}+c.$$

(3) $$t_{1/2}=\;\ln(2)/b.$$

We demonstrate that a relatively higher concentration of VIP leads to a more pronounced reduction in PER2::LUC signals (Figure 3c‐i,ii), and a slow wash‐out of VIP results in a longer duration of the deep trough (Figure 3c‐iii,iv). We calculated the magnitude and rate of PER2 reduction during VIP application at different concentrations (Figure S6a). At three separate VIP applications, the reduction rate of PER2 was 4852.5 ± 1015.1 count/(min*h) under 6 µM VIP, and significantly higher at 13336.7 ± 5652.2 count/(min*h) under 60 µM VIP. We also quantified the magnitude and rate of PER2 restoration during the washout phase (Figure S6b). The rate of PER2 increase following VIP removal was 4311.2 count/(min*h) at a washout flow rate of 0.6 µL/min, and markedly increased to 35411.7 count/(min*h) at 2.5 µL/min.

Such rapid VIP‐induced PER2::LUC signal attenuation does not appear to be influenced by the timing of the 60 µM VIP pulses. It occurs regardless of whether the pulses are administered around peak or trough phases, or other phases within the circadian period (Figure 3a, left panel of Figure 3d). The time lag between the induced trough of PER2::LUC signals and the VIP administration time eventually approaches a steady value of 2 h after two to three cycles (right panel of Figure 3d). We calculated the rate of PER2 reduction in response to VIP stimulation applied at different circadian phases. After two to three cycles, the reduction rates converged to a similar value (Figure S6c). We simultaneously calculated the dependence of both phase delay and magnitude of reduction on the phase of VIP administration (Figure S6d), revealing that PER2 reduction induces a rapid phase‐resetting effect and occurs regardless of the circadian phase at which VIP is applied.

We further validated this VIP‐induced PER2::LUC reduction in engineered MAF cells that ectopically express VIPR2 (Figure 3e), allowing us to pin down the effect of the VIP/VIPR2 pathway in regulating the intracellular clock. As expected, the VIPR2‐free MAF cells (i.e., wt control) show no reduction of PER2::LUC signal, while VIPR2‐containing MAF cells show a reduction similar to the SCN in the presence of 60 µM VIP pulses. These results confirm that the PER2::LUC reduction is directly caused by the VIP signaling pathway's actions within the cellular milieu. Additionally, we compared WT MAF cells and MAF cells with VIPR2 that were initially in the same circadian phase, examining their phase shifts in response to VIP pulses administered at the same time (Figure S7). Only the protein‐expressing cells exhibited a decrease in PER2 signal.

To investigate further the downstream signaling mechanism of VIP/VIPR2 pathway that may mediate the PER2 signal decrease, we treated the brain slices with 30 µM forskolin [23, 24], a compound that directly activated the G_s_‐mediated cyclic AMP (cAMP) signaling pathway [25] through adenylate cyclase and facilitate PER2 expression. Only the anticipated induction, not the rapid reduction of PER2::LUC signals is observed (Figure S8), suggesting that the rapid signal reduction in response to exogenous VIP cannot be attributed to the conventional G_s_‐cAMP signaling cascade. We then explored the potential role of the G_i/o_‐mediated signaling pathway in the rapid reduction of PER2::LUC signals by perfusing pertussis toxin (PTX), a selective G_i/o_ inhibitor (Figure 3f; Figure S9) that could cause attenuated rhythmicity of SCN [26, 27]. The rapid reduction of PER2::LUC signals in response to 60 µM VIP pulses is maintained in the presence of PTX, suggesting that the VIP‐induced PER2 reduction mechanism operates independently of the G_i/o_‐mediated signaling pathway. Moreover, continuous and periodic administration of VIP at high concentrations maintains robust oscillations with consistent amplitude over time (Figure 3f) in the presence of PTX, which was considered to damp the amplitude of oscillation, suggesting that high‐concentration VIP exerts a more substantial influence on regulating PER2 expression than the G_i/o_‐mediated signaling pathway. In conclusion, the rapid decrease of PER2 triggered by VIP pulses extends beyond the cAMP‐dependent negative feedback loop, implicating an alternative mechanism through which VIP modulates circadian rhythm dynamics in SCN neurons.

### 2.4 Cellular Assay for PER2 Reduction

**FIGURE 4:**
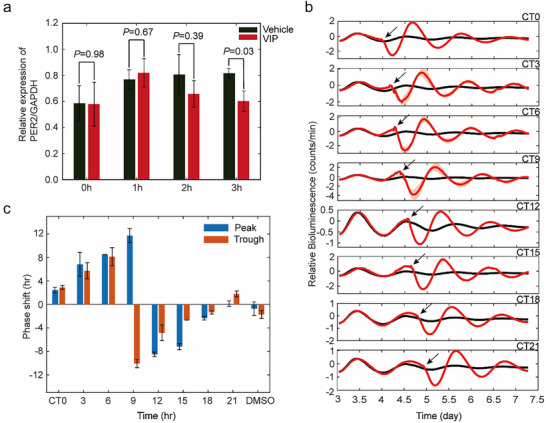
VIP induced PER2 reduction, and related phase and amplitude modulation with cellular assay. (a) HEK293T cells overexpressing VIPR2 were treated with 60 µM VIP, and PER2 protein levels were quantified by western blot. The results are presented as mean ± SE (*n* = 4). (b) PER2‐LUC U2OS cells were co‐transfected with CMV‐PER2‐AID and CMV‐TIR, and treated with 500 µM IAA at different circadian times. Bioluminescence was continuously recorded following IAA addition. Control cells were PER2‐LUC U2OS cells transfected with CMV‐TIR alone, which received 500 µM IAA at either CT0 or CT12. The first bioluminescence trough after recording onset was defined as circadian time zero (CT0). IAA was administered at 3‐h intervals from CT0 to CT21. Data are presented as mean ± SEM (*n* = 3 per time point; control groups *n* = 6, with *n* = 3 for CT0 and CT12). The timing of IAA application is indicated by arrows in the figure. (c) Phase shift of PER2 rhythm of cells with different IAA adding time compared with a regular rhythm. Phase shift data are presented as mean ± SEM.

The swift reduction of PER2::LUC signals, coupled with highly synchronized amplitude and phase of oscillations among individual SCN neurons, implies that this event may potentially link to the direct accumulation and reduction of PER2 protein in the cytoplasm rather than regulation inside the nucleus. To validate our hypothesis, we engineered the VIP signaling pathway in 293T cells by expressing the VIPR2. Following treatment with 60 µM VIP, we found a significant reduction in PER2 protein expression by the third‐hour poststimulation (Figure 4a and Figure S10), indicating that the rapid decrease of PER2::LUC signals is a direct consequence of VIP‐induced PER2 protein reduction. Then we studied the substantial capability of PER2 reduction to regulate the circadian phase by using a U2OS cell line expressing PER2::LUC, incorporating an auxin‐induced degron (AID) domain [28] and TIR1, an E3 ubiquitin ligase adaptor. Once indole‐3‐acetic acid (IAA) is added, the plant hormone auxin can rapidly induce the degradation of PER2‐AID protein in a time‐dependent manner, thereby reducing PER2 abundance in U2OS cells (Figure S11).

Cells expressing LUC‐AID along with the TIR1 were initially analyzed. The LUC‐AID fusion protein is rapidly degraded upon the introduction of IAA (Figure S12). Then U2OS cells expressing PER2‐AID and TIR1 were treated with IAA, and rapid degradation of PER2 and enhanced amplitude of oscillations were observed. (Figure 4b). The induced rapid degradation of PER2 leads to a significant phase shift in a strong Type 0 phase‐resetting manner and amplitude modulation compared to the control group, which is also impacted by the IAA dosing time (Figure 4c), especially when applied at the ascending phase of the rhythm. These cellular assays affirm that the VIP‐induced rapid reduction of PER2 can affect the phase and amplitude of circadian rhythms and even reset the circadian rhythm.

### 2.5 Mathematical Modeling to Mimic the Rapid PER2 Reduction

**FIGURE 5:**
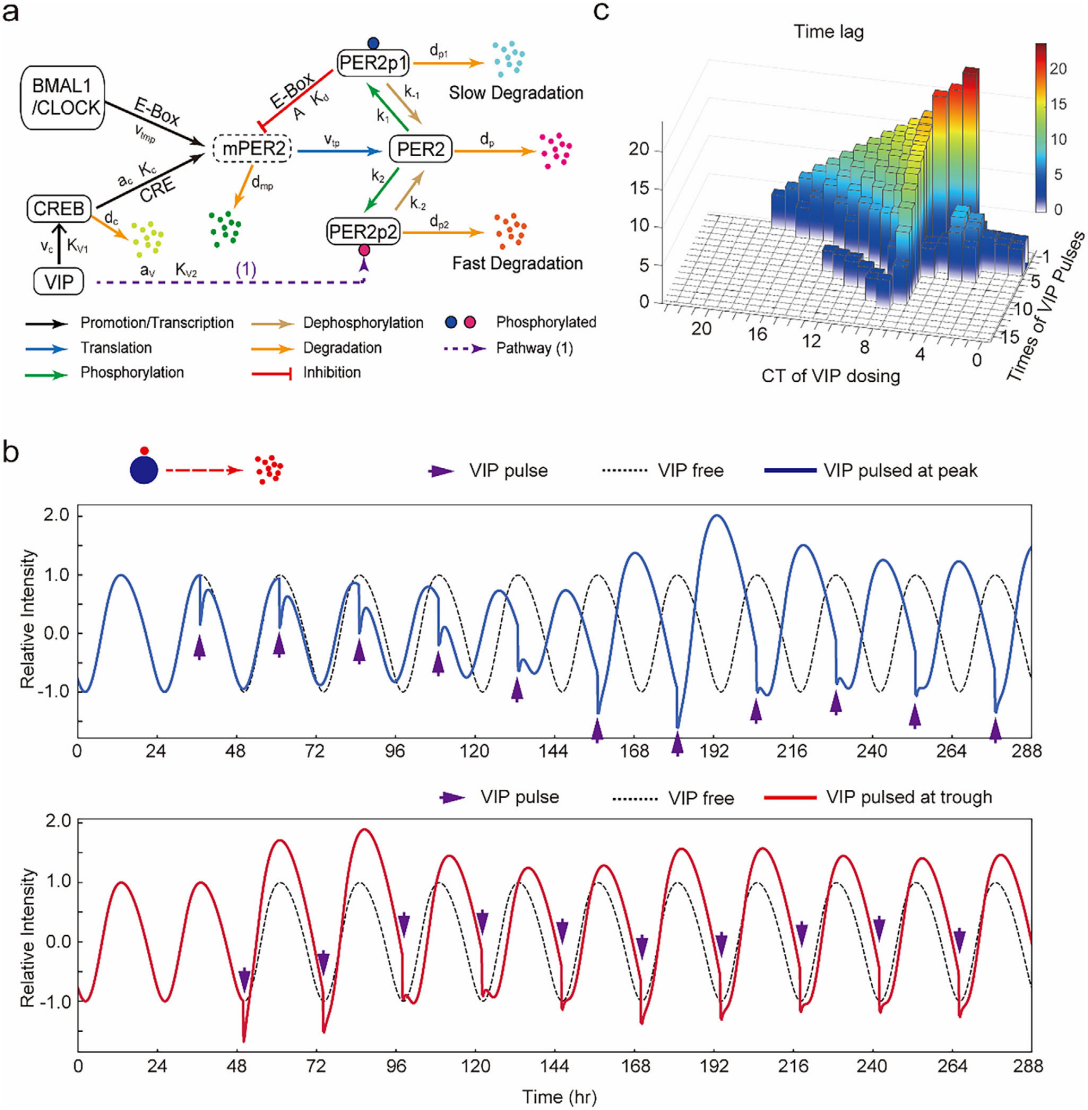
Mathematical modeling of rapid PER2 reduction. (a) Schematic diagram of the circadian clock model incorporating the PER2 negative feedback loop, PER2 phosphorylation at different sites, and VIP signaling pathway. (b) Simulation of the PER2 rhythm with 24‐h‐period VIP pulsing with an assumption that VIP accelerates the degradation of PER2 phosphorylated for fast degradation (blue: VIP initially pulsed at peak; red: VIP initially pulsed at trough). The amplitudes are normalized. (c) Dependence of time lag between VIP pulsing time and induced trough time of PER2 rhythm on the circadian times (CTs) of VIP dosing and times of VIP pulses.

We developed a simplified mathematical model that primarily focuses on PER2 protein‐centric signaling pathways to reconstruct the PER2 circadian rhythm patterns, considering the slow degradation and fast degradation processes of PER2 protein phosphorylated at different sites [29, 30, 31, 32, 33] (Figure 5a). Given the highly synchronized behavior of individual SCN neurons, modeling at the single‐cell level would be sufficient to represent population‐wide outcomes accurately. The principal signaling pathways in our model are articulated using ordinary differential equations. We proposed a potential VIP‐pulse‐triggered signaling cascade, which could expedite the degradation process, assuming that VIP pulses accelerate PER2 protein degradation through phosphorylation, resulting in rapid reduction.

We initially calculated PER2 protein levels without the hypothesized VIP pathway to assess its capacity for simulating PER2 circadian rhythms. Regular intrinsic VIP‐free PER2 oscillations exhibit a period approximating 24 h (Figure S13). As VIP pulsing over four cycles, an augmented PER2 expression is simulated (Figure S13). Subsequently, the model incorporating the hypothesized pathway simulates the circadian rhythm of PER2 as applying VIP at either the peak or trough phase of the oscillation with a 24 h interval. The results demonstrate the strong and instantaneous occurrence of rapid degradation of PER2 in conjunction with VIP pulses (Figure 5b). Gradual phase resetting ensues with each VIP pulse. The amplitude is re‐determined as well. Noteworthy, VIP dosing at peak resets the circadian phase with relatively long periods, while VIP dosing at trough makes troughs swiftly align with the timing of VIP pulses (Figure 5c). As VIP pulse is administered around peak experimentally (Figure 3a), the phase and amplitude reset are completed much faster than the simulated curve (Figure S14), which may be caused by the complex context in SCN slices. Once VIP induces the fast PER2 degradation, regardless of the circadian time at which VIP pulses are applied, the time lag ultimately reaches near zero, and the phase is finally reset. A phase response curve was calculated under periodic VIP pulses as VIP was applied during the rising phase (Figure S15), which behaves very differently compared to the single‐pulse condition, and stabilizes at a relatively low value. The result demonstrates that the rapid degradation of PER2 induced by VIP plays a pivotal role in regulating the phase and amplitude, and resetting the circadian rhythm.

To further investigate the underlying mechanism of PER2 reduction—specifically, whether it results from reduced PER2 production or increased degradation—we performed the simulations using our model by considering VIP accelerating the degradation of mPER2 RNA (Figure S16). Though the phase of oscillation is shifted, no fast degradation of PER2 is observed. Specially, the time of troughs induced by VIP pulses is delayed, while it is consistent with the time of VIP pulses itself as considering fast degradation of PER2, which agrees perfectly with the experimental observations.

The PER2 phosphoswitch model [33], where phosphorylation at the β‐TrCP recognition site leads to fast PER2 degradation, may provide a plausible explanation for our observed phenomenon. The underlying mechanism warrants further detailed experimental exploration, potentially involving a novel signaling pathway activated by VIP.

**FIGURE 6:**
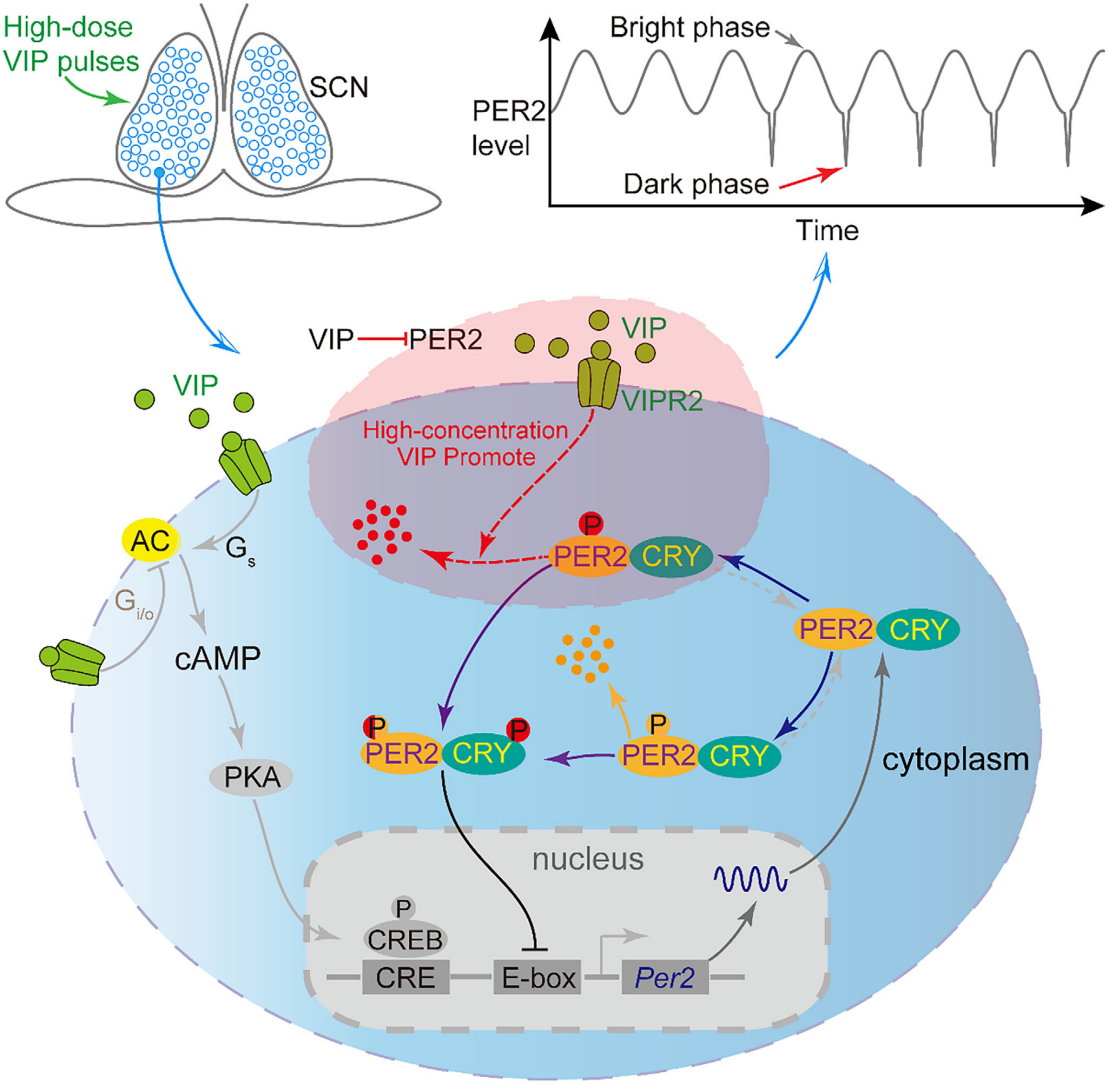
Signaling pathway involved in resetting of a circadian rhythm with high‐concentration VIP induced fast PER2 reduction/degradation.

Building upon the experimental and theoretical evidence outlined, we put forth a model (Figure 6) to elucidate the underlying mechanisms of the observed phenomenon. In high concentrations, VIP rapidly initiates the reduction/degradation of PER2 as the auxiliary mechanism of G_s_ and G_i/o_ mediating signaling pathways. This expedited reduction occurs via PER2 phosphorylation, or an alternative mechanism, which effectively curtails the accumulation of PER2 proteins in the cytoplasm. Consequently, this rapid turnover of PER2 leads to resetting of the circadian rhythm, including the phase and amplitude. Furthermore, we propose a generalized model for circadian phase‐resetting: a fast relief of repressor(s) from the inhibitory complex, such as the reduction/degradation of PER2 or dissociation of another inhibitory protein CRY from the chromatin [34], leads to a drastic phase shift, signifying the crucial role of post‐translational regulation in adjusting the timing of circadian network.

## 3 Discussion

Our experimental findings suggest that sustained and periodic high‐concentration VIP pulses act as a universal resetting signal for the circadian rhythms in SCN neurons. The synchronized reduction in circadian phase‐associated bioluminescence following VIP pulses implies a profound impact on the coupling status of individual neurons within the SCN network, further underscoring the intricate interplay between VIP signaling and clock gene expression in the maintenance and modulation of circadian oscillations. Moreover, the recurring VIP stimuli have been shown to redefine the SCN's circadian time structure by modulating the duration of daytime and nighttime phases, transitioning from a typical 12‐12 day‐night rhythm to an altered ratio closer to 20 h of daytime activity followed by 4 h of nighttime quiescence (Figure 2e). This reconfiguration of the diurnal‐nocturnal period ratio effectively resets the phase of the circadian clock, further substantiating the regulatory impact of VIP on circadian rhythms by manipulating the balance between the length of the active and resting phases.

Upon VIP treatment, PER2 levels were reduced across the cell population and converged toward a similar phase, resulting in synchronized amplitude and phase among cells. Although the molecular mechanism underlying VIP‐induced PER2 reduction remains unclear, the rapidly degrading PER2 using the AID system, which induced a strong Type 0 phase‐resetting response, and a mathematical model considering the rapid PER2 degradation may explain how VIP is capable of driving phase shift and resetting the circadian rhythms when applied at different circadian times.

The bath application failed to elicit the PER2 reduction that we observed in perfusion experiments, as well as the first cycle of the second panel in Figure 3d. This may be caused by the external factors such as temperature shifts and changes in culture medium—particularly serum shock—can significantly alter PER2 expression levels. BaSIC system has minimized strong confounding factors such as temperature fluctuations and serum‐shock effects, allowing us to detect rapid PER2 dynamics that may be obscured under standard conditions. Furthermore, some VIP applications did not produce as pronounced a reduction in PER2::LUC intensity. This variability may be associated with changes in the synchronization level of the cellular network, and it typically coincides with a deterioration in cellular synchrony.

Previous studies have established that the VIP axis functions as a key cellular pacemaker within the SCN [35], and its circadian effects require ERK1/2 signaling as well as coordinated activation of adenylate cyclase and phospholipase C [36, 37]. In our study, applying 60 µM VIP to SCN slices induced a rapid decrease in PER2 and a robust phase shift, which may reflect the rapid VIP release triggered by light stimulation, thereby synchronizing the SCN network—consistent with prior demonstrations that VIP neurons respond rapidly to light [38, 39]. Future work should focus on defining the physiological concentration range and temporal dynamics of endogenous VIP in the SCN, as well as how VIPR2 sensitivity is modulated under different VIP conditions. These factors are essential for fully understanding how VIP reshapes SCN oscillations. Importantly, the BaSIC system developed here provides a powerful tool for dissecting neuropeptide‐mediated circadian regulation with high temporal precision.

In conclusion, we have successfully designed and implemented BaSIC, a modular device, in OSC and circadian studies. This system offers exceptional spatial and temporal resolution, allowing for in‐depth analysis of circadian rhythms within SCN neurons and their molecular mechanisms. Utilizing BaSIC, we have documented the unprecedented observation of VIP‐induced rapid PER2 reduction, an event that not only leads to synchronized behavior in SCN neurons but also resets the circadian rhythm. We have further developed a simplified mathematical model to shed light on the molecular basis of this novel phenomenon. The discovery of clock resetting through VIP‐induced PER2 reduction in mammalian cells has potential implications for clinical study and the treatment of circadian rhythm disorders. This ‘brain‐slice‐in‐a‐chamber’ model paves the way for innovative exploration into the molecular underpinnings of various brain functions, especially those involving air‐liquid interfaces such as in the case of brain organoid cultures.

## 4 Experimental Section

### 4.1 SCN Sample Preparation

Mice were bred and maintained in the National Institute of Biological Sciences (NIBS) specific‐pathogen‐free facility in accordance with the Guide for the Care and Use of Laboratory Animals of NIBS. All experimental procedures were conducted following protocols approved by the Administrative Panel on Laboratory Animal Care (NIBS2024M041) at the National Institute of Biological Sciences (NIBS) in Beijing, China.

SCN slices were prepared according to a previously published protocol [17]. Male or female Per2::luc and Vip‐/‐;Per2::luc mice aged 2–4 months were sacrificed between ZT6 and ZT12. Coronal hypothalamic slices (250 µm thick) containing the SCN were cut using a Leica VT1000 S vibratome and placed onto semi‐permeable membranes (PICM01250, MILLICELL) within 15 min, which were then transferred into culture medium.

The culture medium for SCN slices was the mixture of a pack of DMEM (C11965500BT, Gibco), 20 mL 50x B27 supplement (17504044, Gibco), 4.7 mL 7.5% NaHCO3 (25080094, Thermo Fisher), 10 mL HEPES solution (15630‐080, Gibco), 2.5 mL 10,000 U/mL Penicillin‐Streptomycin (15140122, Gibco), 3.5 g D‐glucose (G8769, Merck), and 800 mL sterile double‐distilled water. The pH of the medium was adjusted to 7.2 using NaOH or HCl solutions, and sterile double‐distilled water was added to make the total volume to 1 L. Additionally, 0.1 mM D‐luciferin (115144‐35‐9, GOLDBIO) was added. Finally, the medium was sterilized by filtration.

### 4.2 Drug Preparation

A synthetic version of mature VIP with 28 amino acid residues was obtained (QYAOBIO). VIP concentration used in the experiments is 60 µM. Forskolin (HY‐15371) and PTX (HY‐112779) were purchased from MedChem Express (MCE). Forskolin and PTX concentrations used in the experiments are 30 µM and 1 µg/mL.

### 4.3 PDMS Spacer Preparation

The PDMS spacer was fabricated using RTV 615 A and B with a mixing ratio of 10:1. The polyether‐ether‐ketone (PEEK) knob stood upside down to a silicon wafer coated with a thin layer of SU8‐2005 (∼5 µm), then mixture was poured into the knob with thickness of 5–8 mm. After degassing, the wafer and knob were put into oven together at 80°C for 30 min. After baking, knob with PDMS spacer was separated from the silicon wafer.

### 4.4 SCNs Experiments and Signal Acquisition

The system temperature is measured using a Pt100 thermocouple located near the sample chamber, and the temperature signal is fed back to an external PID temperature controller to precisely maintain the temperature around 36.5°C (within a specified tolerance of 0.1°C). The volume below the membrane within the BaSIC device is around 200 µL. Two syringe pumps from Harvard Apparatus were used for perfusing different medium and stimuli. Pumps were controlled by custom LabVIEW program. The flow of culture medium is precisely controlled by syringe pump. Exogenous VIP was perfused for 2–4 h, at rate of 2.5 µL/min approximately and then washed out every 24 h automatically. Medium flows at rate of 0.6–1.0 µL/min approximately for the rest time. Medium exchanging with flow rate at 2.5 µL/min for 2 h is long enough to replace the whole volume in a slowly changing way of the culturing conditions. Meantime, the medium flowing rate at 0.6 µL/min is very low, which cannot change the internal temperature in the culture chamber but keeps a constant nutrition. Both the temperature and the syringe pump flow rate have been calibrated and are kept stable throughout the measurement process. Forskolin experiments used the same perfusing parameter. For PTX experiments, medium with 1 µg/mL PTX was added to replace the regular culture medium after a period of measurement. VIP pulses used the same perfusing parameter.

Bio‐luminescent signals taken by PMTs (Hamamatsu Photonics) were collected individually using a NI PXI system with photon counting module (National Instruments), controlled through custom LabVIEW program. Images were taken using an Andor ikon CCD (M‐934) (Oxford Instruments) through 10X objective lens, which operated at −90°C with a water‐cooling system. A strategic 700 nm cut‐off filter (Thorlabs China) preceded the CCD, shielding against environmental noise, particularly the infrared light sources. All systems ran automatically. The exposure time for each image was around 30 min, with a binning setting of 2 × 2 pixels.

### 4.5 Data Analysis of Images

The bioluminescence data were analyzed with ImageJ and a custom MATLAB program. After images were collected, a pretreatment was applied with ImageJ to remove outliers. A pixel binning setting of 2 × 2 was employed for enhanced signal‐to‐noise ratio. The final pixel size is 26 µm Í 26 µm. Then, a custom MATLAB program was used to process the data of every pixel in the images.

We defined the synchronization index (*SI*) to quantify phase synchronization across pixels in the images. We used the average circadian rhythm as a reference, and calculated the phase shift for each pixel. We denoted the phase shift of j^th^ pixel as δϕ_j_. Then we computed *SI* with the following equation:

(4) $$SI=\frac{1}{n}\;\sum_{j\;=1}^{n}e^{i\delta \phi_{j}}$$

In Equation (4), *i* denotes imaginary unit, and *n* denotes the number of pixels. We calculated *SI* (0 ≤ *SI* ≤ 1) at each rhythmic peak and trough, where larger *SI* represented larger degree of synchronization.

### 4.6 Engineering 293T Cells

VIPR2 protein was expressed in 293T cells (CRL‐1573, ATCC) by transient transfection. The VIPR2 plasmid and lipo3000 (L3000015, Invitrogen) were diluted with Opti‐MEM (31985070, Gibco), respectively. Then the two components were mixed together and incubated at room temperature for 5 min. The 293T cell suspension was added to the mixture, and cells were incubated at 37°C for 2 days. Then the transfected cells were passaged into the 12‐well plate, and cultured until they reached more than 90% confluence. VIP was added to the medium to achieve the final concentration of 60 µM. Cells were collected at 0 h, first h, second h, and third h post‐treatment, then washed with PBS and frozen in liquid nitrogen. The collected samples were thawed and added to SDS loading buffer diluted with lysate, then boiled at 100°C for 15 min. Samples were mixed and centrifuged at maximum speed for 2 min, and the supernatant was collected for verification with a Western blot.

### 4.7 Western Blot

Protein samples were separated by SDS‐PAGE, and then transferred to a polyvinylidene difluoride membrane. The membrane was blocked with skim milk for 2 h at room temperature to prevent non‐specific binding. It was then incubated overnight at 4°C with the primary antibodies against PER2 (PER21‐A, Alpha Diagnostic International) and GAPDH (ab8245, Abcam). The following day membranes were incubated with species‐appropriate secondary antibodies conjugated to horseradish peroxidase: anti‐mouse IgG or anti‐rabbit IgG (4408S, Cell Signaling Technology). The protein bands were visualized using Immobilon ECL substrate (WBULS0100, Millipore). Cells were treated with high concentrations of VIP and sampled at 0‐, 1‐, 2‐, and 3‐h poststimulation. GAPDH levels were used as a loading control.

### 4.8 Engineering PER2::LUC U2OS cells carrying PER2‐AID‐HA and TIR1‐myc

PER2::LUC U2OS cells (HTB‐96, ATCC) expressing TIR1‐myc served as a negative control. *Per2* and *Aid* genes were synthesized by Azenta. Human *Per2* and *Aid* DNA fragments were generated using PCR amplification with primers containing a 20 bp overlap, and then assembled into a pCDH‐CMV‐MCS‐EF1‐GFP‐T2A‐Puro plasmid (CD510B‐1, System Biosciences). TIR1 DNA fragments were generated using PCR amplification and then assembled into a pCDH‐CMV‐MCS‐EF1‐RFP‐T2A‐Puro plasmid (CD516B‐2, System Biosciences). Lentivirus was prepared in HEK293T cells by co‐transfection of transfer plasmid with helper plasmids VSV.G (14888, Addgene), pRSV‐Rev (12253, Addgene) and pMDLg (12251, Addgene). The virus‐containing supernatant was collected 44 h post‐transfection, filtered through a 0.45 µm filter, and used to infect U2OS cells seeded at 20% confluence.

### 4.9 Bioluminescence Recording of PER2‐AID System

PER2::LUC U2OS cells carrying PER2‐AID‐HA and TIR1‐myc, along with control cells, were seeded at a density of 90% in 3.5 cm Petri dishes. After allowing the cells to adhere to the bottom of the dishes, the culture medium was replaced. Cells were then transferred into a Lumicycle (Actimetrics) for bioluminescence recording. Approximately 20 h later, when the PER2::LUC bioluminescence curve reached its trough, IAA (500 µM, I2886, Sigma‐Aldrich) was added to both cell lines at different time points throughout the circadian cycle, with treatments spaced 3‐h interval. Bioluminescence was recorded for 4 days, and the data were analyzed using Lumicycle Analysis software.

### 4.10 Cells Experiments in BaSIC

We generated PER2::LUC MAF cells overexpressing VIPR2, as well as MAF cells co‐expressing VIPR2 and CMV‐driven luciferase. The procedures for viral packaging and infection followed the same workflow described above for *Engineering PER2::LUC U2OS cells carrying PER2‐AID‐HA and TIR1‐myc*. MAFs were carefully cultured on the bottom glass of BaSIC, providing an ideal environment for firm adhesion and the establishment of proper cellular functions. The attachment process occurred at 37°C and lasted between 2 h and 8 h. Following the adhesion phase, BaSIC was meticulously assembled with external perfusion pumps, setting the stage for real‐time and controlled experimentation. Throughout the experimental phase, the culture medium was perfused at a steady rate of 0.5 µL/min for a 21‐h period, ensuring a stable micro‐environment for the cells. Subsequently, a VIP solution was introduced at a higher flow rate of 1.5 µL/min for 3 h. This manipulation of flowing rates served dual purposes: minimizing the serum shock experienced by the cells and maintaining their adherence to the glass substrate during the experiment. The PMT system was used to record the signals.

### 4.11 Mathematical Modeling

*mPER2*, *PER2*, *PER2p1*, *PER2p2*, and *CREB* were time‐dependent variables in our mathematical model. Pathways considered in our model included *Per2* transcription and translation, phosphorylation and dephosphorylation events, protein degradation, and VIP signaling pathways. For simplifying the process, the phosphorylation of PER2 resulting in slow degradation was condensed into a single‐step event rather than multiple sequential steps. We built ordinary differential equations (Equations (5), (6), (7), (8), (9), (10)) of these pathways and solved the time‐dependent function of above variables. The value of *V* was set before solving the equations. The initial values of variables were listed in Table S1.

(5) $$\begin{array}{ccc} \frac{d\left[mPER2\right]}{dt} & = & v_{\mathrm{tmp}}\;\left(1+a_{c}\frac{{\left[CREB\right]}^{2}}{K_{c}^{2}+{\left[CREB\right]}^{2}}\right)\\ & & \times \;f\left(\left[PER2p1\right],\;A,\;K_{d}\right)-d_{\mathrm{mp}}\left[mPER2\right] \end{array}$$

(6) $$\begin{array}{ccc} \frac{d\left[PER2\right]}{dt} & = & v_{\mathrm{tp}}\;\left[mPER2\right]+k_{-1}\left[PER2p1\right]+k_{-2}\left[PER2p2\right]\\ & & -\left(k_{1}+k_{2}\right)\left[PER2\right]-d_{p}\left[PER2\right]\; \end{array}$$

(7) $$\begin{array}{ccc} \frac{d\left[PER2p1\right]}{dt} & = & k_{1}\;\left[PER2\right]\\ & & -\;k_{-1}\left[PER2p1\right]-d_{p1}\left[PER2p1\right] \end{array}$$

(8) $$\begin{array}{ccc} \frac{d\left[PER2p2\right]}{dt} & = & k_{2}\;\left[PER2\right]-k_{-2}\left[PER2p1\right]\\ & & -\;d_{p2}\left(1+a_{V}\frac{V^{2}}{K_{V2}^{2}+V^{2}}\right)\left[PER2p2\right] \end{array}$$

(9) $$\frac{d\left[CREB\right]}{dt}=v_{c}\;\frac{V^{2}}{K_{V1}^{2}+V^{2}}-d_{c}\left[CREB\right]$$

(10) $$\begin{array}{c} f\;\left(x,\;A,\;K_{d}\right)=\frac{1}{2}\;\left(1-\frac{x}{A}-\frac{K_{d}}{A}+\sqrt{{\left(1-\frac{x}{A}-\frac{K_{d}}{A}\right)}^{2}+\frac{4K_{d}}{A}}\right) \end{array}$$

In Equation (5), $v_{\mathrm{tmp}}\left(1\;+\;a_{c}{\left[CREB\right]}^{2}/\left(K_{c}^{2}\;+{\left[CREB\right]}^{2}\right)\right)f\left([PER2p1],A,\;K_{d}\right)$ denoted *PER2* gene transcription, which promoted by *CREB* and inhibited by phosphorylated *PER2*. Function *f* (Equation 10) represented the inhibition of *PER2* transcription by phosphorylated *PER2*. d_mp_[*mPER2*] denoted *PER2* mRNA degradation. In Equation (6), v_tp_[*mPER2*] denoted *PER2* translation. In Equations ((6), (7), (8)), k_−1_[*PER2* *p*1] and k_−2_[*PER2* *p*2] denoted *PER2* dephosphorylation, while k_1_[*PER2*] and k_2_[*PER2*] denoted *PER2* phosphorylation. d_p_[*PER2*], d_p1_[*PER2* *p*1], and d_p2_[*PER2* *p*2] denoted the degradation of *PER2*, *PER2p1*, *PER2p2*, respectively. *PER2p2* degradation was accelerated by VIP, which was reflected by $\left(1+a_{V}V^{2}/\left(K_{V2}^{2}+V^{2}\right)\right)$. In Equation (9), $v_{c}\;V^{2}/\left(K_{V1}^{2}+V^{2}\right)$ denoted activation of CREB by VIP, and d_c_[*CREB*] denoted degradation of *CREB*. The values of parameters were shown in Table S2.

### 4.12 Statistical Analysis

MATLAB was used to analyze experimental data, including preprocessing of data, generating plots, and calculating sample statistics. For data preprocessing, we smoothed PER2::LUC rhythm data and removed the baseline by VMD algorithm. Then we normalized PER2::LUC rhythm data, so that we were able to calculate period, amplitude, and phase of PER2 rhythm. Western blot and IAA‐treated cell data are presented as mean ± SEM. N replicates were quantified in Western blot experiment, and 3 replicates were analyzed in IAA‐treated cell experiment. For statistical comparison of independent samples, the two‐sided tests were used, with *p* < 0.05 as the threshold for significance.

## Author Contributions

K. H., M. L., and J. Z. contributed equally to this work and shared the first authorship. Y. H. and E. E. Z. initiated the concept and supervised the project. K. H. designed the optical and fluidic experiments. K. H. and R. Z. designed the BaSIC module. K. H. contributed to the construction of the entire BaSIC system, including the hardware and software. K. H. and M. L. conducted SCN and single‐cell experiments. M. L. contributed to all the SCN and cell lines preparations for the experiments. L. M. generated the MAF cells harboring *Vipr2*. M. L. conducted Western blot experiments. K. H. and J. Z. performed image analysis and data analysis. J. Z. and K. H. built the mathematical model. Y. H., E. E. Z., K. H. M. L., J. Z., and D.J. wrote the paper with inputs from all authors.

## Conflicts of Interest

The authors declare no conflicts of interest.

## Supporting information




**Supporting File**: advs73865‐sup‐0001‐SuppMat.docx.

## Data Availability

The data that support the findings of this study are available from the corresponding author upon reasonable request.
