# Supplementary material for: An Automated Organotypic SCN Culture System Revealing Novel Insights into VIP Regulation of Circadian Rhythm
Source: Adv Sci (Weinh). 2026 Jan 15;13(10):e11069. doi: 10.1002/advs.202511069 (PMC12915189; doi:10.1002/advs.202511069)
Supplement: Supplementary file 1 — Supporting File: advs73865‐sup‐0001‐SuppMat.docx. [file ADVS-13-e11069-s001.docx]

**Supporting Information**


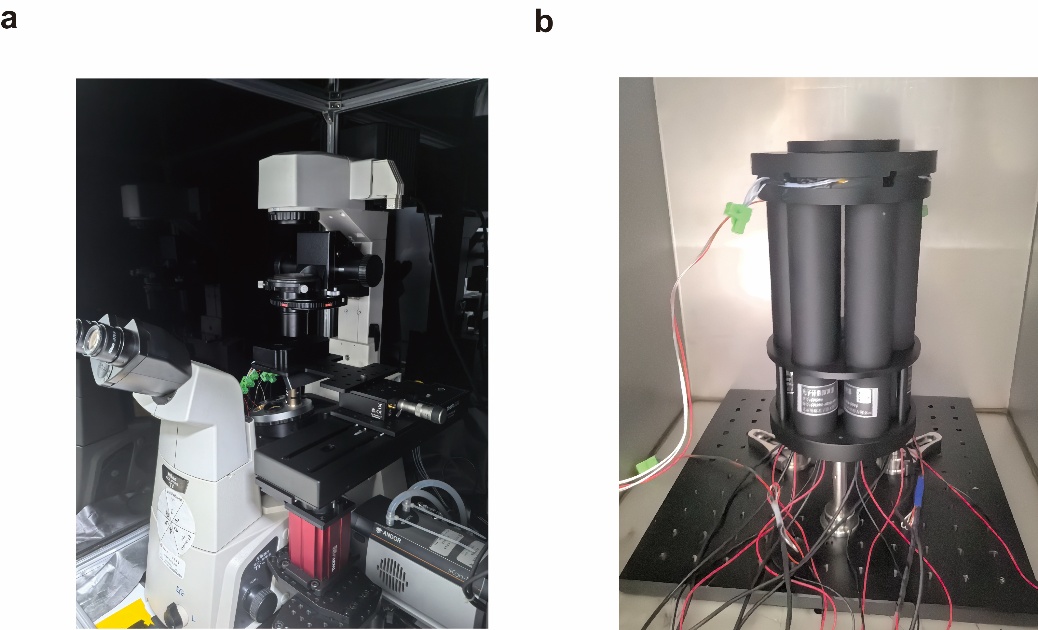


**Figure S1 |** **Experimental setup**. **a**, imaging system comprising a microscope and a CCD camera to study circadian rhythm of SCN. BaSIC devices housed in a customed holder is positioned on the microscope stage for imaging. **b**, parallel system featuring PMTs supporting up to six independent channels for studying collective rhythms. This system accommodates six BaSIC units placed within a customed holder, enabling simultaneous data acquisition across all channels. The entire setup is controlled using LabVIEW programs, which operate both hardware control and data acquisition.


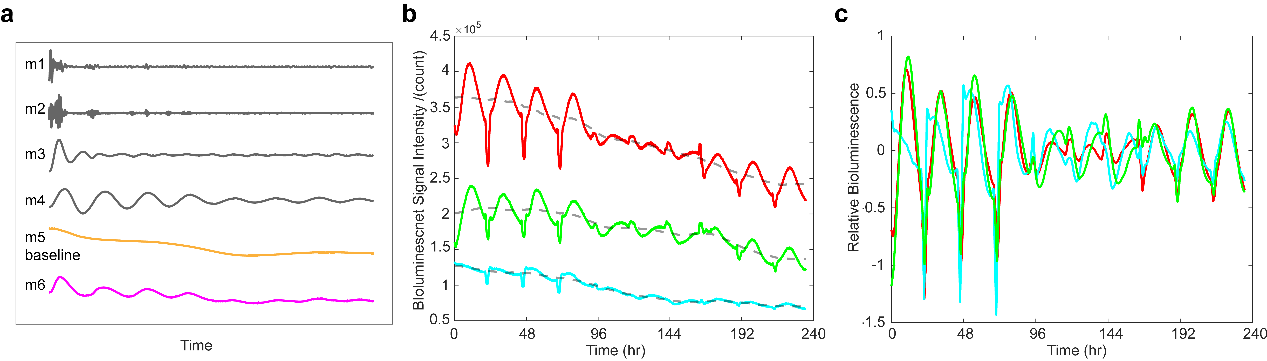


**Figure S2 | VMD analysis of experimental data.** **a**, different modes (m1-m5) are calculated by VMD, representing modes with different frequencies, while m6 represents the original curve. Among these, m5, which has the lowest frequency, is selected as the baseline. **b**, the red, green, and blue solid curves represent original data collected from parallel experiments, whereas the dashed lines indicate the baseline calculated with VMD. The baseline calculation performed with VMD demonstrates robustness across different rhythm curves. **c**, the red, green, and blue curves shown here represent rhythm curves after background subtraction.


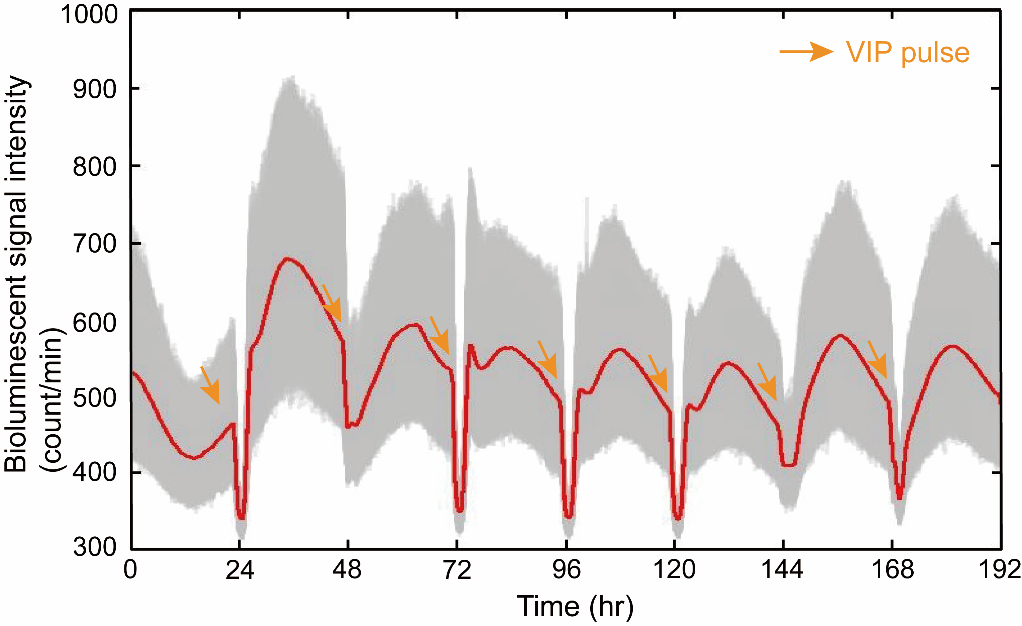


**Figure S3 | Time-domain PER2::LUC bioluminescent signals of each pixels in SCN area.** The grey curves represent the circadian rhythms of individual pixels, while the red curve indicates the average rhythm across all pixels. Orange arrows denote the time points of VIP stimulations, which are administered at 24-hour intervals. These VIP stimulations induce rapid decrease of the PER2::LUC signals, and the circadian rhythms of the cells in the SCN are highly synchronized by the VIP pulses.


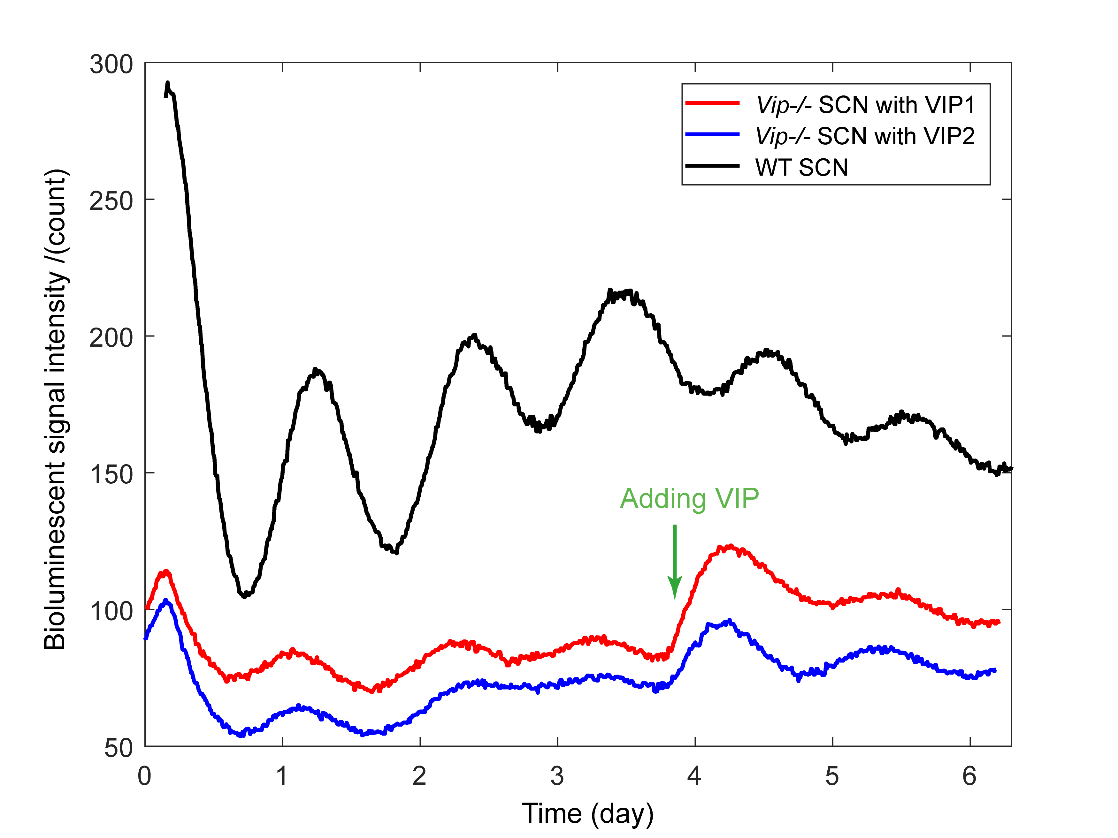


**Figure S4 | VIP-stimulated SCN samples in dishes.** 60 µM VIP from different suppliers (VIP1 and VIP2) was manually added to 35mm dishes within LumiCycle apparatus. The red and blue curves represent the time-dependent PER2::LUC signals in *Vip-/-* SCN treated with VIP1 and VIP2 pulses, respectively. The time point of VIP stimulation is marked with green arrow. The black curve shows the time-dependent PER2::LUC signal in wild-type SCN. Notably, we did not observe the rapid decrease in the PER2::LUC signals following VIP stimulations in the Petri-dish experiments.
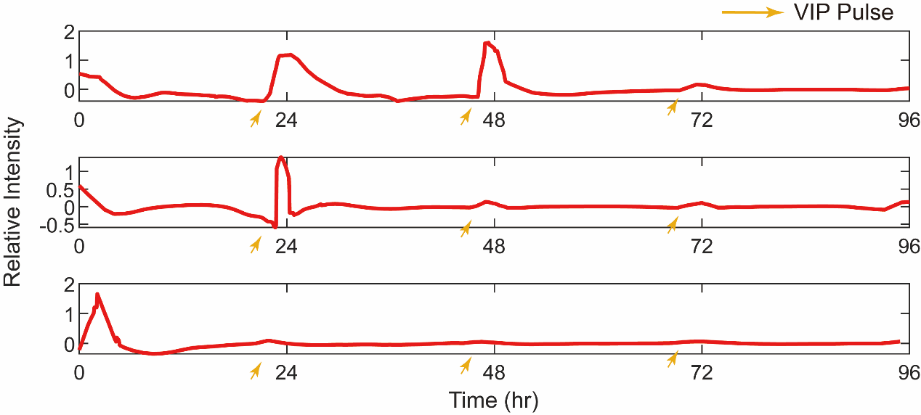


**Figure S5 | 60 µM VIP pulses treated engineered MAF cell line expressing CMV-LUC.** MAF cells cultured in BaSIC and measured with the PMT system. VIP pulses are marked with orange arrows. Only the peak induced by serum shock was observed, with no reduction in Luciferase signal.


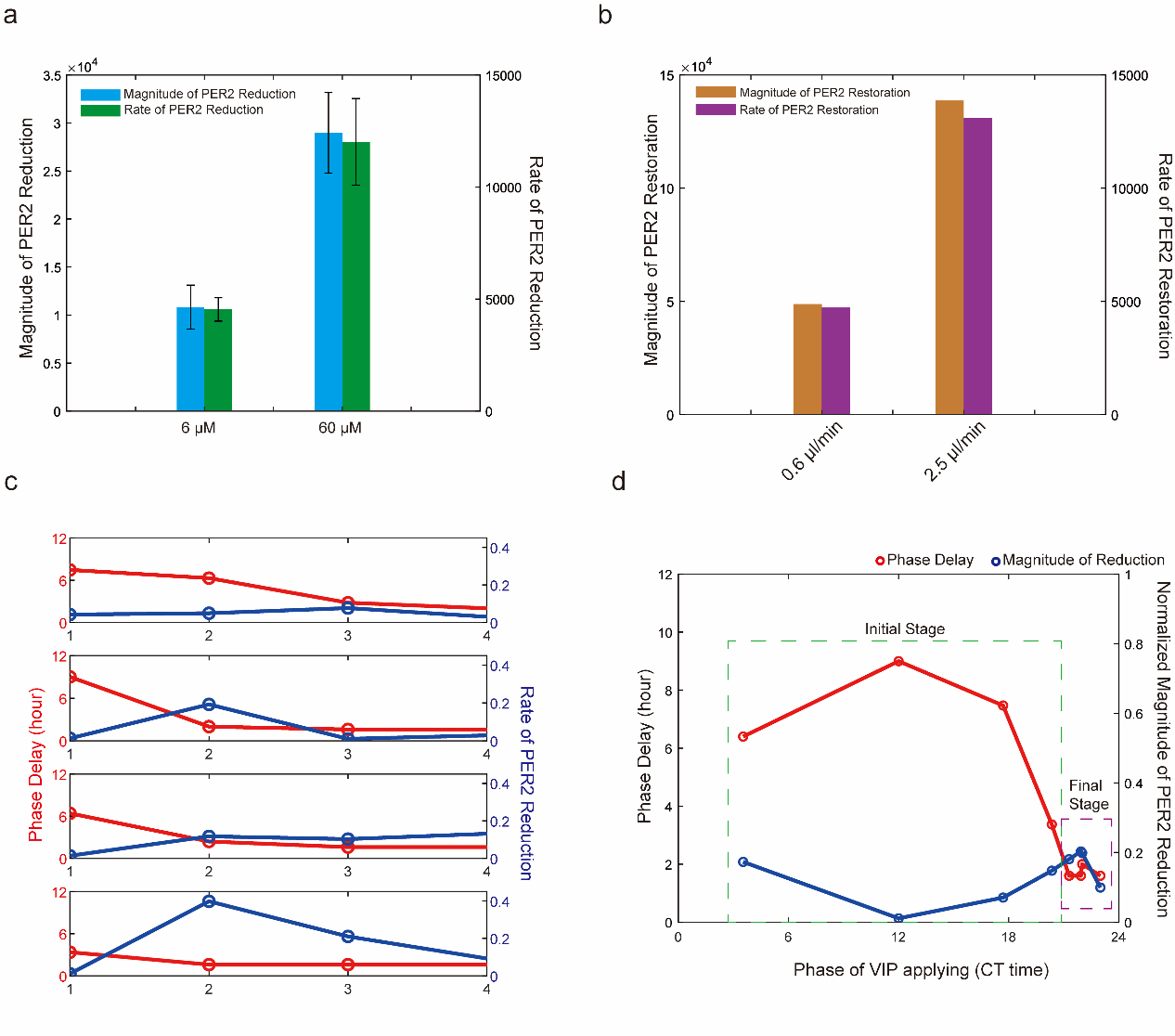


**Figure S6 | Analyses of the relationships between VIP concentration, washout rate, application timing, and their respective effects on PER2 dynamics.** **a**, magnitude (blue) and rate (green) of PER2 reduction in *Vip-/-* SCN slices during stimulation with 6 μM and 60 μM VIP. **b**, magnitude (orange) and rate (purple) of PER2 restoration in *Vip-/-* SCN slices following washout of 60 μM VIP. **c**, phase delay induced by VIP (red) and rate of PER2 reduction (blue) in four *Vip-/-* SCN slices subjected to four 24-hour-period VIP pulses administered at different phases of the PER2 rhythm. **d**, phase delay induced by VIP (red) and magnitude of PER2 reduction (blue) in four *Vip-/-* SCN slices after four doses of VIP, plotted against the circadian phase of VIP application.


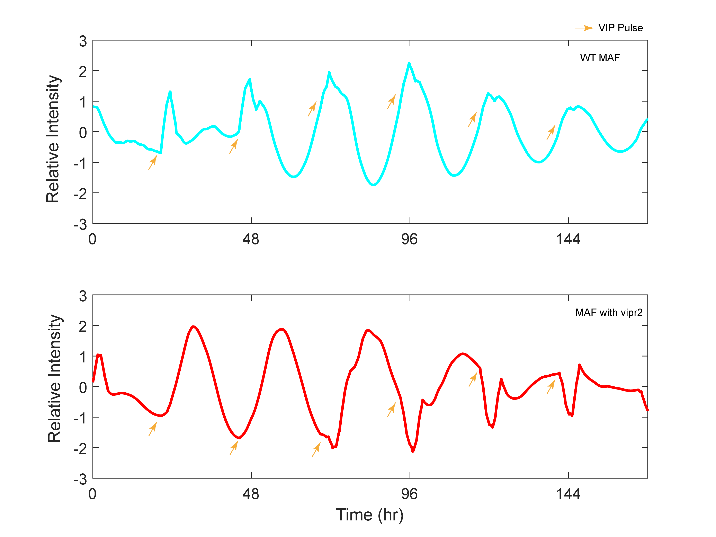


**Figure S7 | Cells response to VIP applying at the same phase.** When 60 µM VIP was applied at the same circadian phase, WT cells exhibited no response other than the transient signal increase caused by serum shock, whereas Vipr2-expressing cells displayed a rapid, transient decrease in PER2-LUC signal.


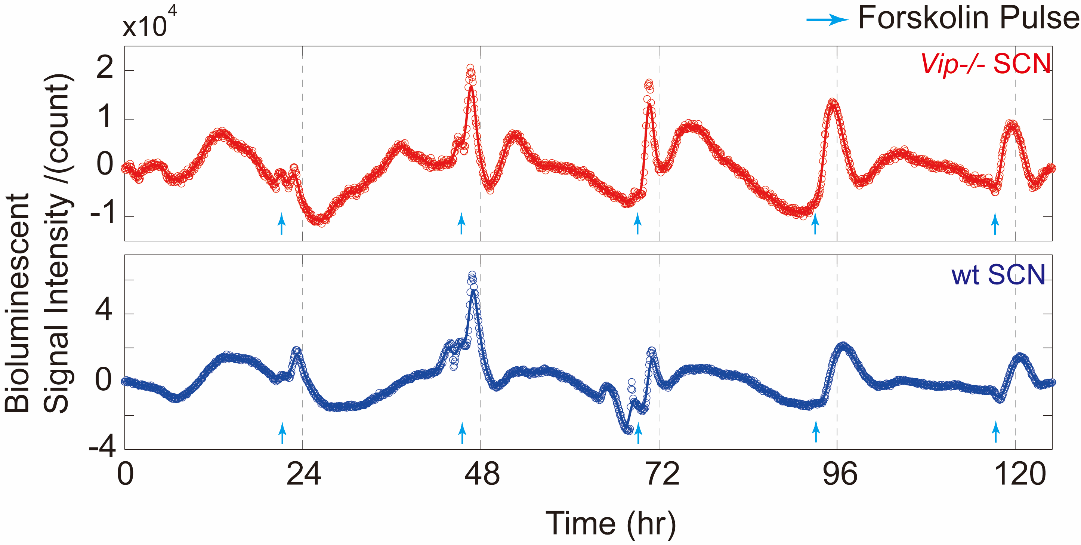


**Figure S8 | Periodical Forskolin pulses stimulated SCNs in BaSIC.** The red curve represents the circadian rhythm of *Vip-/-* SCN with 30 μM forskolin stimulations administered at 24-hour intervals, while the blue curve depicts the circadian rhythm of wild-type SCN under the same of forskolin stimulation protocol. The time points of forskolin pulses are indicated by blue arrows. Notably, forskolin stimulations do not induce the rapid decrease in the PER2::LUC signals.


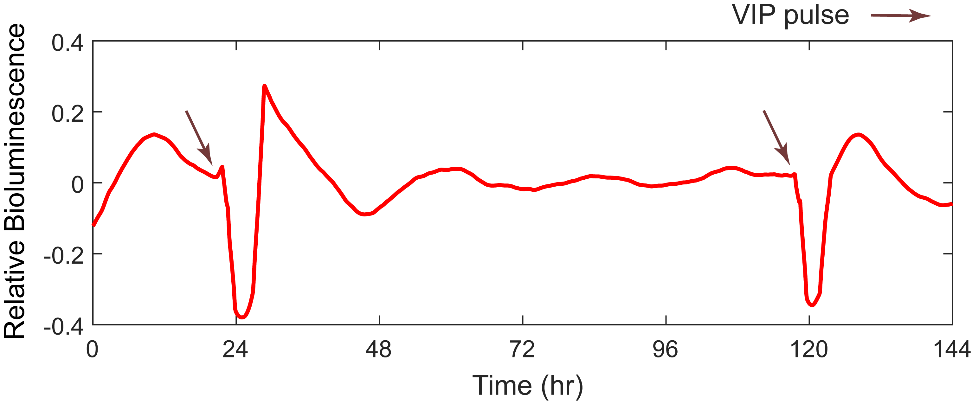


**Figure S9 | VIP-induced oscillation damping by PTX.** The red curve represents the circadian oscillation of the wild-type SCN. The SCN slice was cultured in a medium containing PTX between two pulses of VIP stimulation. The presence of PTX leads to rapid damping of the circadian oscillation, but it does not affect the decrease in the PER2::LUC signal triggered by VIP.


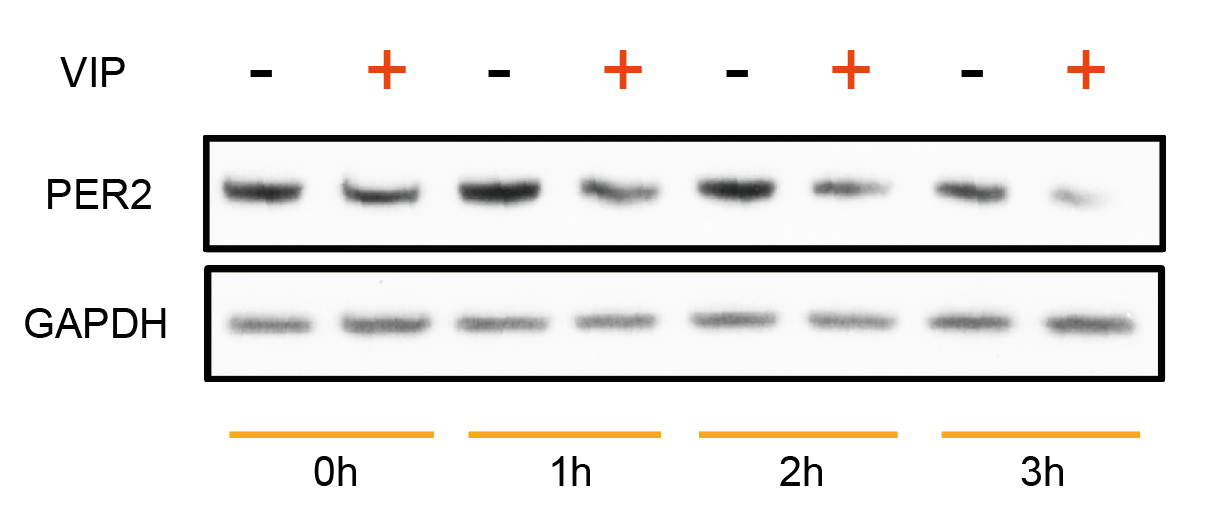


**Figure S10 | PER2 protein levels after 60 µM VIP treatment by western blot.**


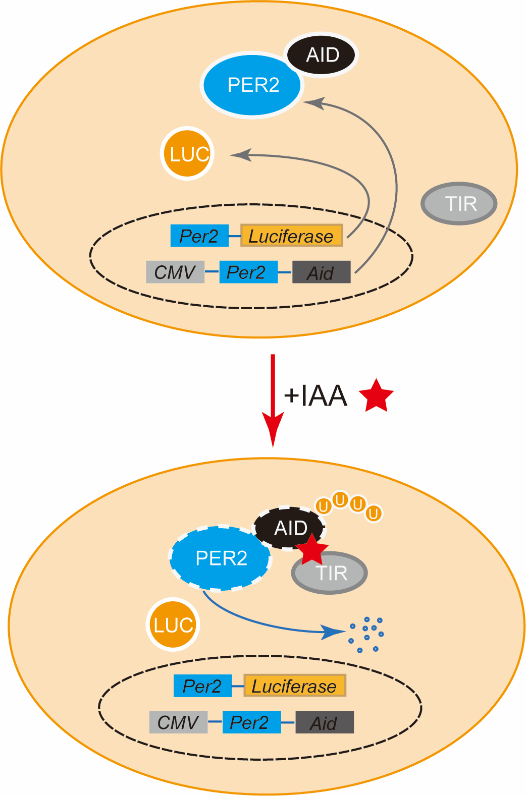


**Figure S11 | Engineering strategy of the U2OS cell line with PER2::LUC and PER2-AID-TIR1 system.** We employed synthetic biology method to engineer U2OS cell line expressing PER2::LUC by incorporating an auxin-induced degron (AID) domain and TIR1 (an E3 ubiquitin ligase adaptor). Once adding indole-3-acetic acid (IAA), the plant hormone auxin can rapidly induce the degradation of PER2-AID protein in a time-dependent manner, thereby disrupting the clock gene expression in U2OS cells.


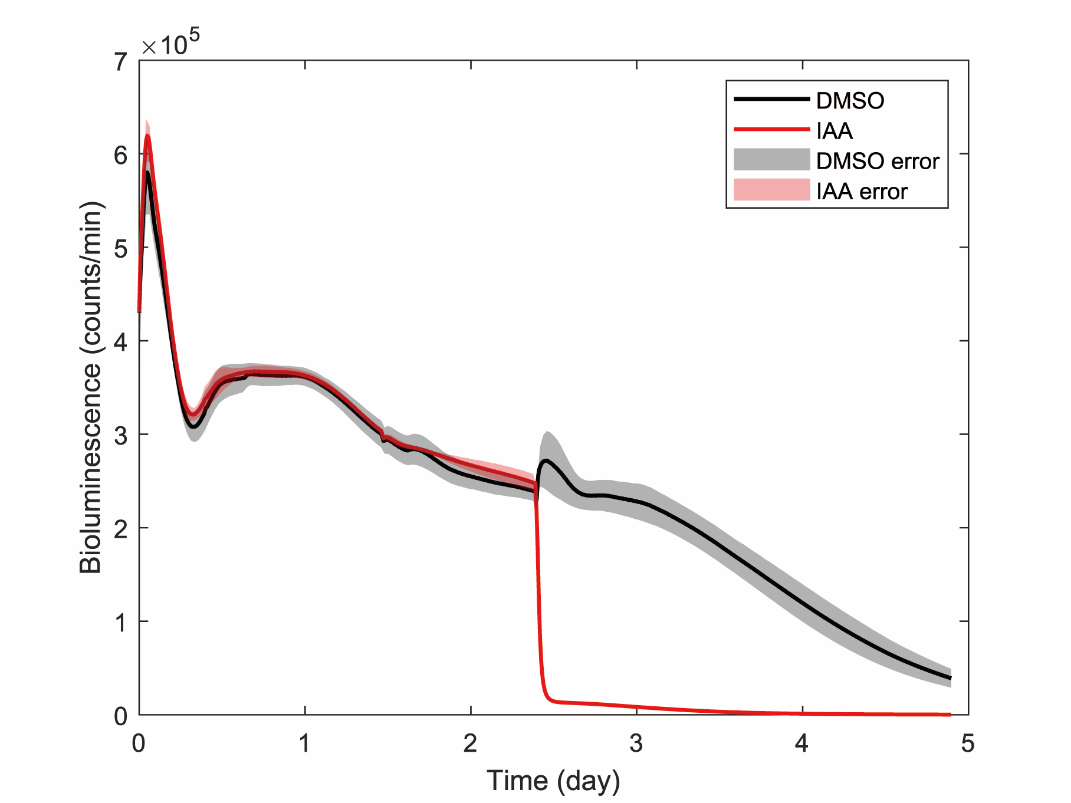


**Figure S12 | Result of IAA degrading the CMV-LUC-AID system.** The red curve represents the averaged LUC signal from parallel experiments with IAA treatment at 2.5 day. The black curve shows the averaged LUC signal from parallel experiments with DMSO treatment at 2.5 day, serving as the control. The shaded areas indicate the standard deviation. After the addition of IAA, the LUC signal rapidly decreases, suggesting that LUC degradation is stimulated by IAA.


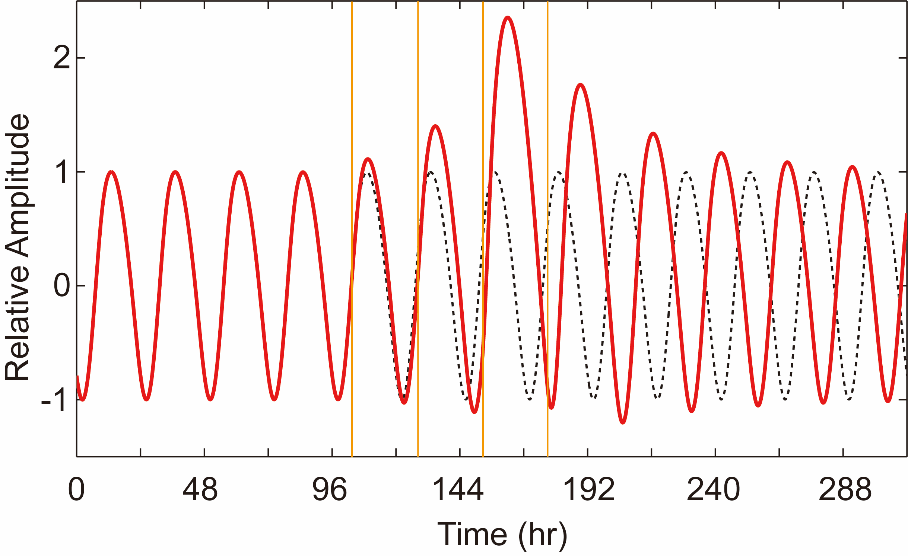


**Figure S13 | Simulated circadian oscillations of PER2**. The black dash line represents the normal PER2 oscillation of a single neuron in SCN, while the red line indicates the circadian oscillation of PER2 with VIP pulses without the hypothesized pathways. The times at which VIP was added are marked with orange lines. Without the hypothesized pathways, VIP pulses do not cause a rapid decrease of PER2 in the circadian oscillation.


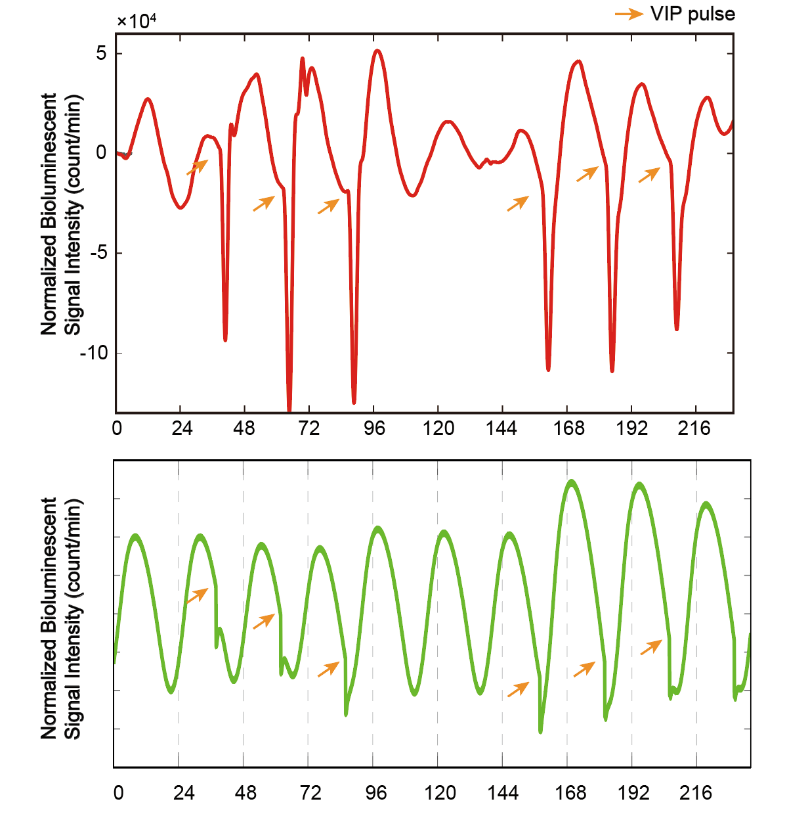


**Figure S14 | Simulation result in green using experimental parameters according to Figure 3 (a). VIP pulses are marked with orange arrows.**


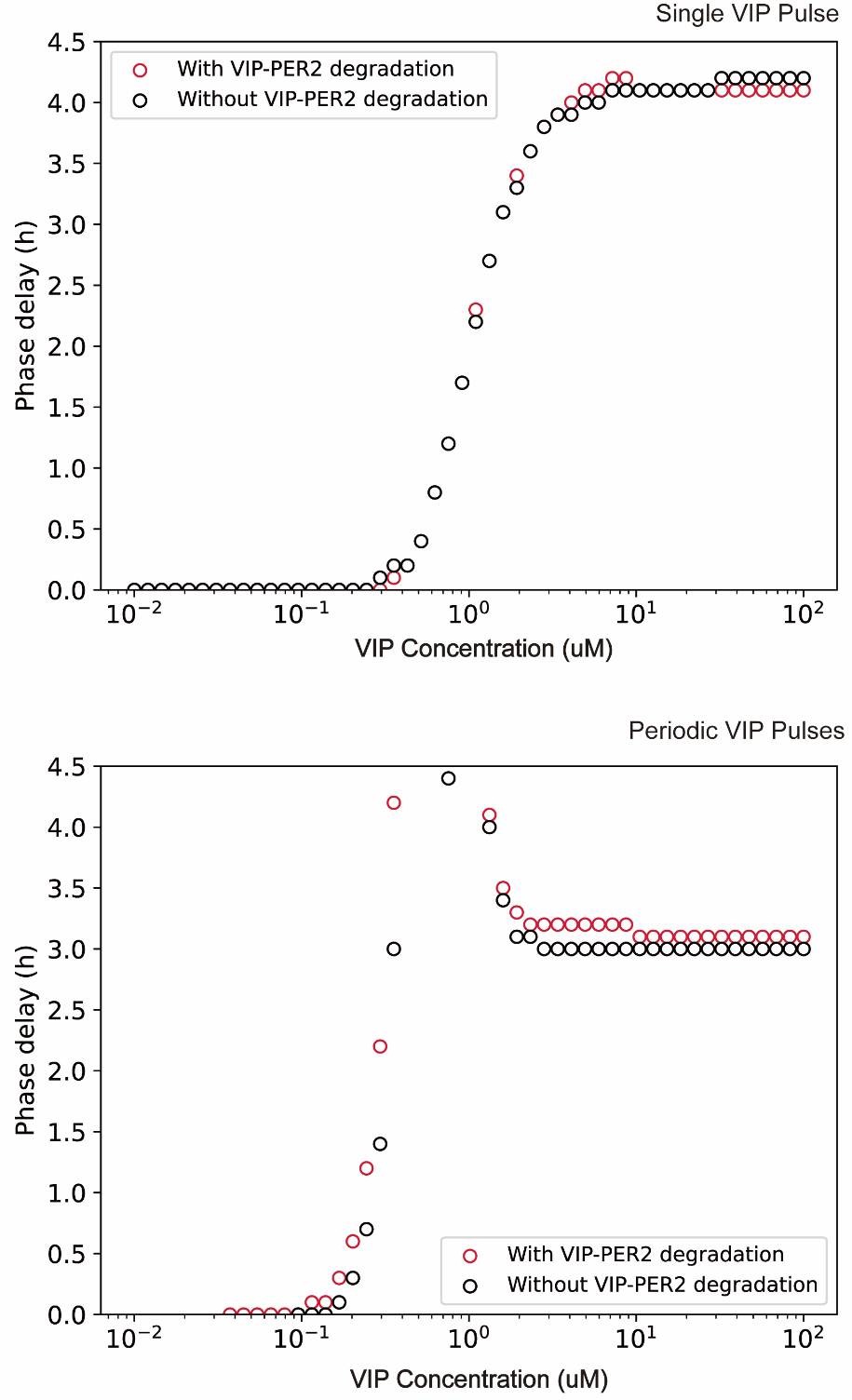


**Figure S15 | Phase response curves both in the case of a single VIP pulse and periodic VIP pulses.** The resulting PRC differs slightly depending on whether rapid PER2 protein degradation is assumed. The phase delay induced by periodic VIP pulses behaves very differently at high concentrations compared to the single-pulse condition, which stabilized at a relatively low value.


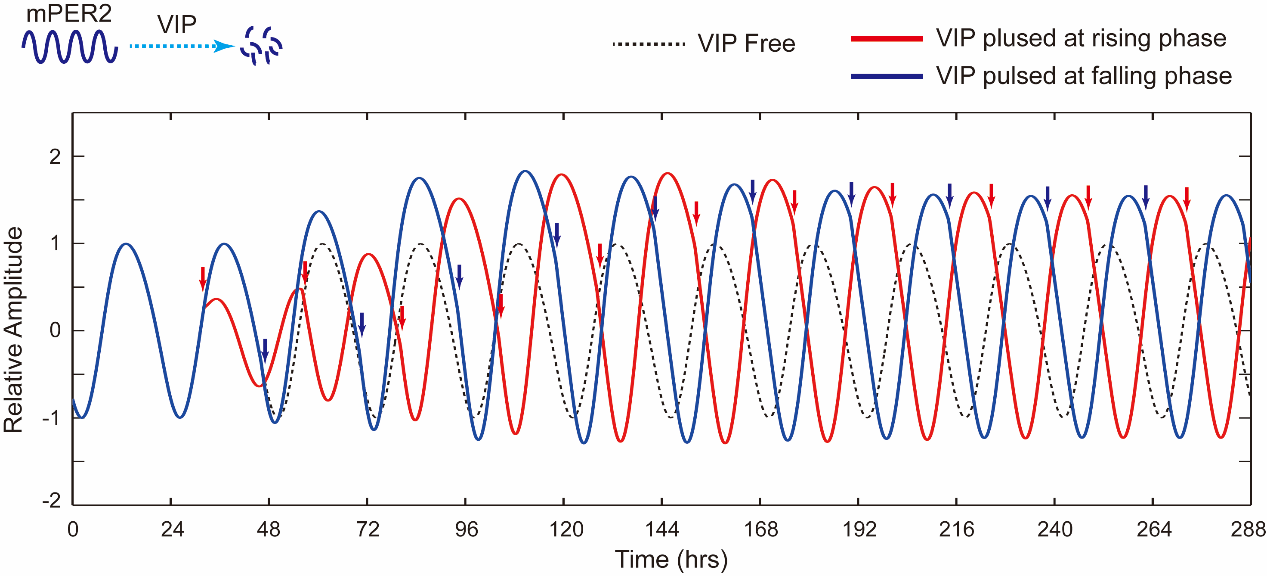


**Figure S16 | Simulation result of mathematical model by considering VIP accelerating the degradation of mPER2 RNA.**

**Table S1:** Variables in the model

| Index | Variable | Description | Initial Value | Unit |
| --- | --- | --- | --- | --- |
| 1 | *mPER2* | The concentration of Period2 mRNA | 0.386 | nM |
| 2 | *PER2* | The concentration of Period2 protein | 0.241 | nM |
| 3 | *PER2p1* | The concentration of Period2 protein phosphorylated on the FASP site | 1.33 | nM |
| 4 | *PER2p2* | The concentration of Period2 protein phosphorylated on the β-TrCP site | 0.0558 | nM |
| 5 | *CREB* | The concentration of activated cAMP response element-binding protein | 0 | nM |
| 6 | *V* | The concentration of vasoactive intestinal peptide | 0 | nM |

**Table S2:** Parameters in the model

| Index | | Parameter | | Description | Value | Unit | Reference |  |
| --- | --- | --- | --- | --- | --- | --- | --- | --- |
| 1 | | v_tmp_ | | Maximal transcription rate for *Per2* mRNA | 2.8 | nM/h | Estimated |  |
| 2 | | v_tp_ | | Translation rate constant for PER2 | 0.1 | 1/h | Estimated |  |
| 3 | | v_c_ | | Activation rate for CREB | 1.2 | nM/h | Estimated |  |
| 4 | | k_1_ | | Phosphorylation rate constant for PER2 at FASP site | 0.5 | 1/h | Estimated |  |
| 5 | | k_−1_ | | Dephosphorylation rate constant for PER2 at FASP site | 0.1 | 1/h | Estimated |  |
| 6 | | k_2_ | | Phosphorylation rate constant for PER2 at β-TrCP site | 0.2 | 1/h | Estimated |  |
| 7 | | k_−2_ | | Dephosphorylation rate constant for PER2 at β*-*TrCP site | 0.04 | 1/h | Estimated |  |
| 8 | | d_mp_ | | Degradation rate constant for *Per2* mRNA | 0.075 | 1/h | Estimated |  |
| 9 | | d_p_ | | Degradation rate constant for PER2 | 0.0225 | 1/h | Estimated |  |
| 10 | | d_p1_ | | Degradation rate constant for phosphorylated PER2 at FASP site | 0.00084 | 1/h | Estimated from ^2^ |  |
| 11 | | d_p2_ | | Degradation rate constant for phosphorylated PER2 at β-TrCP site | 0.833 | 1/h | Estimated from ^2^ |  |
| 12 | | d_c_ | | Degradation rate constant for CREB | | 0.125 | 1/h | Estimated |
| 13 | | a_c_ | | Promotion coeﬀicient for CREB on *Per2* transcription | | 2.0 | none | Estimated |
| 14 | | K_−2_ | | Hill constant for promotion of CREB on *Per2* transcription | | 0.5 | nM | Estimated |
| 15 | | K_d_ | | Dissociation constant between PER2 and BMAL1-CLOCK | | 1.555*×*10*^−^*^5^ | nM | ^2^ |
| 16 | | A | | Total BMAL1-CLOCK concentration in nucleus | | 1.366 | nM | ^2^ |
| 17 | | K_V 1_ | | Hill constant for promotion of VIP on CREB activation | | 1 | nM | Estimated |
| 18 | | a_V_ | | Promotion coeﬀicient for VIP on the degradation of PER2 phosphorylated at β-TrCP site | | 12 | none | Estimated |
| 19 | | K_V 2_ | | Hill constant for VIP on degradation of PER2 phosphorylated at *β*-TrCP site | | 0.5 | nM | Estimated |
| 20 | | a_Vm_ | | Promotion coeﬀicient for VIP on the degradation of *Per2* mRNA | | 12 | none | Estimated |
| 21 | | K_V 3_ | | Hill constant for VIP on degradation of *Per2* mRNA | | 0.5 | nM | Estimated |
